# Genetic adaptations in the population history of *Arabidopsis thaliana*

**DOI:** 10.1093/g3journal/jkad218

**Published:** 2023-09-25

**Authors:** Hirohisa Kishino, Reiichiro Nakamichi, Shuichi Kitada

**Affiliations:** Graduate School of Agricultural and Life Sciences, The University of Tokyo, 1-1-1 Yayoi, Bunkyo-ku, Tokyo 113-8657, Japan; Research and Development Initiative, Chuo University, 1-13-27 Kasuga, Bunkyo-ku, Tokyo 112-8551, Japan; Fisheries Resources Institute, Japan Fisheries Research and Education Agency, 2-12-4 Fukuura, Kanazawa-ku, Yokohama, Kanagawa 236-8648, Japan; Graduate School of Marine Science and Technology, Tokyo University of Marine Science and Technology, 4-5-7 Konan, Minato-ku, Tokyo 108-8477, Japan

**Keywords:** *Arabidopsis thaliana*, coadaptation, gene expression adaptation, phenotypic adaptation, population history, Plant Genetics and Genomics

## Abstract

A population encounters a variety of environmental stresses, so the full source of its resilience can only be captured by collecting all the signatures of adaptation to the selection of the local environment in its population history. Based on the multiomic data of *Arabidopsis thaliana*, we constructed a database of phenotypic adaptations (p-adaptations) and gene expression (e-adaptations) adaptations in the population. Through the enrichment analysis of the identified adaptations, we inferred a likely scenario of adaptation that is consistent with the biological evidence from experimental work. We analyzed the dynamics of the allele frequencies at the 23,880 QTLs of 174 traits and 8,618 eQTLs of 1,829 genes with respect to the total SNPs in the genomes and identified 650 p-adaptations and 3,925 e-adaptations [false discovery rate (FDR) = 0.05]. The population underwent large-scale p-adaptations and e-adaptations along 4 lineages. Extremely cold winters and short summers prolonged seed dormancy and expanded the root system architecture. Low temperatures prolonged the growing season, and low light intensity required the increased chloroplast activity. The subtropical and humid environment enhanced phytohormone signaling pathways in response to the biotic and abiotic stresses. Exposure to heavy metals selected alleles for lower heavy metal uptake from soil, lower growth rate, lower resistance to bacteria, and higher expression of photosynthetic genes were selected. The p-adaptations are directly interpretable, while the coadapted gene expressions reflect the physiological requirements for the adaptation. The integration of this information characterizes when and where the population has experienced environmental stress and how the population responded at the molecular level.

## Introduction

In its history of range expansion and migration, a population has experienced biotic and abiotic stresses and has adapted to these stresses by selecting its alleles that can cope with them. In the case of insecticide resistance in *Drosophila*, a single allele saved the population (Daborn et al. 2002). On the other hand, the case of herbicide tolerance in the weedy morning glory revealed the cost of adaptation (Baucom and Mauricio 2004). In plant–pathogen interactions, both plant resistance pathways and pathogen's virulence mechanisms are influenced by environmental factors such as temperature and humidity. The complex nature of adaptation is difficult to capture in experiments with controlled environmental conditions (Velásquez et al. 2018). On the other hand, the phenotypic variance between populations may be either inflated or reduced, depending on the G × E effect. Therefore, the magnitude of phenotypic change across environments is not in itself indicative of the underlying pattern of genotypic influence. Common garden experiments allow the detection of genetic adaptation of traits with countergradient variation (Levins 1969; Conover and Schultz 1995).

As a model plant species, the adaptation of *Arabidopsis thaliana* has been extensively studied for important traits and environments; flowering times (Izawa 2007; Li et al. 2010; Ågren et al. 2017; Zan and Carlborg 2019); seed dormancy (Alonso-Blanco et al. 2003); timing of germination (Zacchello et al. 2020); tolerance to freezing (Ågren and Schemske 2012); response to salt, osmotic, and cold stress (Kreps et al. 2002; Busoms et al. 2021); water-use efficiency (Dittberner et al. 2018); and acclimation to light intensity (Stewart et al. 2015). Although *A. thaliana* is selfing, the linkage disequilibrium decays rapidly, within 50 kb (Nordborg et al. 2005). This indicates the high accuracy of the population genomic inference on the genomic diversity, population structure, and population history. Approximate Bayesian Computation (ABC; Beaumont et al. 2002) with the spatial coalescent model was applied to the above sequence fragments from 76 European individuals and inferred from the longitudinal gradient of genomic polymorphism that the major migration in Europe occurred from the east about 10,000 years ago and spread westward at a rate of about 0.9 km/year (François et al. 2008).

The 1001 Genomes Consortium presented a detailed map of variation in the genomes of 1,135 naturally inbred lines representing the native Eurasian and North African range and recently colonized North America (The 1001 Genomes Consortium 2016). Pair-wise divergence analysis identified 26 outliers, which were referred as relicts (from glacial refugium). Out of the 26 relicts, 22 were in the Iberian Peninsula, and 1 line each from the Cape Verde Islands, Canary Islands, Sicily, and Lebanon. Model-based inference of population structure (Pritchard et al. 2000; Alexander et al. 2009) identified 1 group consisting of relicts and 8 genetic clusters of nonrelicts. While the polymorphism of relicts showed the sign of isolation by distance (IBD), that of nonrelicts did not, possibly because of admixture with relicts. By carefully eliminating the effect of admixture with the relicts, Lee et al. (2017) suggested the Balkans or the Black Sea region as the most likely origin for the spread of the nonrelicts. The widespread relicts were replaced by the nonrelicts, which rapidly spread westward, probably in association with agriculture and human commensal in general. To infer the pattern of introduction into the North American population and adaptation of immigrants, Shirsekar et al. (2021) collected 2,861 individuals from an area of 1,200 by 900 km in the Eastern United States. Their analysis of haplotype sharing with the global Afro-Eurasian collection inferred the ancestry profiles in detail.

In the face of dramatic climate change, it is important to understand how the species will respond to it. To predict the potential in the standing populations of *A. thaliana*, Exposito-Alonso et al. (2017) investigated the genetic variants that are associated with survival in an extreme drought event. The genetic alleles that increased survival showed a signature of polygenic adaptation and were more common in Mediterranean and Scandinavian regions. Recent genetic association studies have shown that most complex traits are polygenic. For example, de Miguel et al. (2022) estimated the degree of polygenicity of fitness-related traits in a long-lived plant, *Pinus pinaster* Ait., maritime pine. The degree of polygenicity ranged from 0 to 27%, with an average of 6%, across traits, environments, and years. To increase the sensitivity of detecting polygenic adaptation via subtle allele frequency shifts at many loci, Berg and Coop (2014) combined the knowledge from genome-wide association studies (GWAS) with robust population genetic modeling. Using the effect size estimates of QTLs, they proposed to correlate the mean genetic value with the environmental variables. They also defined a statistic that measures the excess variance of the mean genetic value between populations compared to that expected due to drift. To detect the polygenic adaptation in the admixture graphs, Racimo et al. (2018) developed a Bayesian model that contrasts the allele frequency dynamics of the QTLs with those of neutral loci.

A population encounters a variety of environmental stresses, so the full source of its resilience can only be captured by collecting all the signatures of adaptation to the selection of the local environment in its population history. The multiomic database generated by the 1001 Genomes Project gave rise to the databases collecting the information on the phenome, transcriptome, and genome associations (Togninalli et al. 2020; Lan et al. 2021). To obtain a global picture of the genetic adaptations in the population history of *A. thaliana*, we constructed in this paper a database of the phenotypic adaptations (p-adaptations) and gene expression adaptations (e-adaptations) from the 1001 Genomes database. Utilizing the above databases, we studied the dynamics of allele frequencies at 23,880 QTLs for 174 traits and 8,618 eQTLs for 1,829 genes over population history. Traits such as the time to flowering and germination rates are quantitative traits, while those such as protist disease resistance and “growing includes September” are 0–1 scored qualitative traits. Here we refer to the SNPs associated with the trait values of both types as QTL. For each trait/gene expression, polygenic p-adaptation/e-adaptation along the edges of the admixture graph (Pickrell and Pritchard 2012) was identified as the set of changes in allele frequencies at its QTLs/eQTLs that are collectively predicted to significantly alter the genetic value of the trait/gene expression (Racimo et al. 2018). Through the enrichment analysis of the identified adaptations, we inferred a likely scenario of adaptation that is consistent with the biological evidence from experimental work. The constructed database of p-adaptations and e-adaptations, combined with the climatic information, can characterize the stress of the local environment. The information on the trait adaptations is directly interpretable. The coadapted gene expressions may reflect the physiological requirements for adaptation to that stress. The integration of both types of the information provides an indication of where and when the *A. thaliana* population experienced the environmental stresses, how it was affected by these stresses, and how it responded to them.

## Materials and methods

### Multiomic data

The 1001 Genomes Project of *A. thaliana* (The 1001 Genomes Consortium 2016) has generated multiomic data (e.g. genome, transcriptome, methylome, and phenome) and has resulted in several GWAS-based databases: Arapheno and AraGWAS Catalog, which contains the genome×phenotype associations (Togninalli et al. 2020), and CLIMtools, which contains the environment×genome×phenotype association (Fick and Hijmans 2017, Ferrero-Serrano and Assmann 2019). AtMAD (Lan et al. 2021) was designed to integrate the information on the associations between genome, transcriptome, methylome, pathways, and phenotype.

The data sets of sampling locations and whole-genome SNP genotypes of the 1,135 *A. thaliana* accessions (Supplementary Fig. 1) were downloaded from 1001 Genomes (The 1001 Genomes Consortium 2016). The data of transcriptome expression values was obtained from NCBI GEO (GSE80744, GSE43858, and GSE54680). The information of the QTLs of the phenotypes and the eQTLs of gene expressions were obtained from AtMAD, which contained all the identified QTLs/eQTLs with *P*-values less than $10^{-6}$. Out of 516 phenotypes in Arapheno, 248 had identified QTLs with *P*-values less than $10^{-6}$. Out of 33,602 gene expressions, 2,879 had identified eQTLs with *P*-values less than $10^{-6}$. Out of 2,879 gene expressions, 1,438 had more than 1 eQTLs. The annotations of QTL/eQTL-associated genes were obtained from the databases of Entrez, GO, and TAIR (Maglott et al., 2011; Berardini et al. 2015, The Gene Ontology Consortium 2020) using R packages rentrez (Winter 2017), AnnotationDbi (Pagès et al. 2023), org.At.tair.db (Carlson 2019), and GO.db (Carlson 2016).

### Estimation of the population history

To estimate the history of range expansion, admixture, and introgression, we applied TreeMix, ver 1.13 (Pickrell and Pritchard 2012), to the set of the current populations of *A. thaliana*. Unlike humans, the wild weed *A. thaliana* has no obvious genetic barriers across the national borders. Misspecification of the population units can lead to large uncertainties in estimating population history. For the population units, we first followed the assignment of the 1,135 accessions to the genetic clusters by ADMIXTURE (*k* = 9, Alexander et al. 2009, The 1001 Genomes Consortium 2016). The genetic clusters consist of 1 relict group and 8 nonrelict groups, broadly corresponding to geography, labeled as Asia, Central Europe (CEurope), Germany, Italy–Balkan–Caucasus (ItaBalCau), Northern Sweden (NSweden), Southern Sweden (SSweden), Spain, and Western Europe (WEurope). To get a global view of local adaptations, we further subdivided them into countries: Armenia (ARM), Austria (AUT), Azerbaijan (AZE), Bulgaria (BUL), Czech Republic (CZE), Spain (ESP), France (FRA), Georgia (GEO), Germany (GER), Italy (ITA), Kyrgyzstan (KGZ), Lithuania (LTU), the Netherlands (NED), Portugal (POR), Romania (ROU), Russian Federation (RUS), Serbia (SRB), Switzerland (SUI), Slovakia (SVK), Sweden (SWE), Tajikistan (TJK), United Kingdom of Great Britain and Northern Ireland (UK), United States of America (USA), and Uzbekistan (UZB). We focused on the populations from which at least 3 individuals were sampled. As a result, 1,073 accessions from 46 populations were included for the analysis. For this analysis, we used whole-genome SNPs filtered by 4 criteria, nonindel, biallelic, missing rate <1%, and allele frequency >1%. The computation time of TreeMix is generally proportional to the number of SNPs. Although TreeMix allows for SNPs with missing values, we chose the threshold 1% of missing rate to maximize the coverage of the SNPs given the computational limit. As a result, the allele frequencies of 37,718 filtered whole-genome SNPs were analyzed (see Supplementary Methods 1 in Supplementary information for the number of SNPs remaining at each stage of filtering). The individual SNP genotypes are available at https://zenodo.org/record/7903201:

TreeMix runs with a specified outgroup and given the number of admixture events. We adopted the set of relicts as an outgroup of the other nonrelicts. These relicts were not sampled from historical sites, but were identified as outlier accessions in the distribution of genetic distances between accessions (The 1001 Genomes Consortium 2016, Supplementary Fig. 2). Regardless of the nomenclature, the set of relicts can be a valid outgroup that is genetically distinct from the nonrelicts.

TreeMix formulates the between-population variance matrix of the allele frequencies by the set of genetic drifts and admixtures. The observed variance matrix with its standard errors was modeled by a multivariate normal distribution and fitted by maximum composite likelihood procedure. We used all of the 37,718 filtered neutral SNPs used for the admixture graph estimation. Since the genome size of *A. thaliana* is $1.35\times 10^{8}$ bp, the average distance of neighboring SNPs is $1.35\times 10^{8}/37,718\;=$ 3,580 bp. The standard errors may be underestimated, if SNPs with LD are assumed to be independent. However, the point estimate of the admixture graph may be unbiased, although the degree of its uncertainty may be underestimated and the composite likelihood value may be overestimated.

We ran the TreeMix for the number of the admixture events from 0 up to 15. The maximum log-likelihood value increased with the number of admixture events, but it was difficult to determine the number of admixtures. This was partly because the graph was estimated by maximizing the composite likelihood. We were not ready to obtain the valid effective degree of freedom to be set as a penalty against the over-parametrization (but see Varin et al. 2011). Following the idea of Evanno et al. (2005), who estimate the number of genetic clusters for the STRUCTURE software, OptM (Fitak, 2021) estimates the number of admixture edges, *m*, based on the ratio, Δ(m), of the second-order rate of increase of the maximum composite likelihood, L(m), to its inter-iteration standard deviation. Although OptM suggested m=1 among the candidate values, m=1,…,15, the variance explained was 93%, far below the value of 99.8% suggested by the authors of TreeMix. The maximum composite likelihood did not reach a plateau either but continued to increase linearly with *m*. Although we set the maximum value of *m* for practical computational reasons for this TreeMix analysis and the downstream PolyGraph analysis of traits and gene expressions, the output of OptM seemed to imply an insufficient range of *m* for comparison. Visual inspection of the admixture graphs showed that the global pattern of nonmigrant edges was qualitatively unchanged across different values of *m*. As a compromise between sufficient fit to the data and the computational feasibility, we adopted the m=10 admixture graph for the subsequent analyses of multitrait and multigene expression coadaptations (see Discussion for more explicit and detailed observations).

### Construction of selection matrices

Adaptation of traits and gene expression was detected by applying PolyGraph (Racimo et al. 2018). For each of the traits/gene expressions, PolyGraph contrasts the variation in allele frequencies at QTLs/eQTLs with that at the neutral SNPs and estimates the selection parameters along the edges of the admixture graph. Considering that each of the variants causing the trait/gene expression polymorphism may have a weak signal, the selection of the variant is not estimated individually, but the mean selection parameter is estimated, assuming the uniformity of the selection across the QTLs/eQTLs. Therefore, the estimated selection parameters of the traits/gene expressions express the directional changes in allele frequencies at the set of QTLs and eQTLs in relation to the trait values and the levels of gene expressions, beyond the level of genetic drift.

The AtMAD database contained 49,973 QTLs and 16,672 eQTLs. We analyzed the trait/gene expression, if any of the QTLs/eQTLs of a trait/gene expression were measured in at least 1 individual in each of the 46 populations. As a result, we analyzed 174 traits out of 248 with identified QTLs and 1,829 gene expressions out of 2,879 with identified eQTLs. The number of QTLs and eQTLs that we analyzed was 23,880 and 8,618, respectively (see Supplementary Methods 2 for detail). The traits and gene expressions that were analyzed by PolyGraph can be found in Supplementary Data 1 and 2, and the annotated lists are available at https://zenodo.org/record/7903201.

The required input of the eQTL effect sizes in PolyGraph were available from the eQTL data set. For the QTLs, we regressed the trait value on the alleles of the QTL, for each trait and for each QTL. To account for the effect of population structure, we included the first 5 principal components of the genotype data of whole-genome SNPs as covariates. We adopted the admixture graph from TreeMix as the required population history input. For the neutral SNPs to be contrasted, we excluded the QTLs/eQTLs of the trait/gene expression from the 37,718 filtered SNPs (Supplementary Methods 1).

We attempted to obtain a global picture of the adaptation in the population history by integrating the p-adaptations of all traits and the e-adaptations of all gene expressions. For each trait and for each gene expression, we obtained its “posterior *P*-values” from the Markov Chain Monte Carlo (MCMC) samples of the selection parameters at the edges of the admixture graph. First, we obtained the *z*-value of the selection parameters as the ratio of the posterior mean and the posterior standard deviation from the MCMC samples. Then, assuming normality of the estimates, we obtained the *P*-values. We constructed matrices of the *z*-values for traits and for gene expressions. The rows of the matrices represent the edges of the admixture graph, and the columns represent the traits and gene expressions. By applying the Benjamini–Hochberg procedure (Benjamini and Hochberg 1995) to each of the matrices, we obtained a map of significant p-adaptations and e-adaptations on the admixture graph.

As a tool to test for polygenic adaptation, Berg and Coop (2014) developed the $Q_{X}$ statistic, which measures the excess variance of the mean genetic value between populations compared to that expected due to drift. Following this idea, Racimo et al. (2018) introduced the $Q_{B}$ statistic to prioritize the edges to be explored in the MCMC. They chose 1/3 of the maximum value of $Q_{B}$ as the cut-off value and ran the MCMC for the edges with $Q_{B}$ greater than this value. Numerical simulation confirmed that the edges with polygenic adaptation were retained in the pre-MCMC screening and that the MCMC correctly identified them, at least for the highly polygenic traits (400 SNPs affecting the traits) under strong selection (selection parameter = 0.2). The *P*-values of the edges excluded in the pre-MCMC screening were set to 1. Our testing procedure seemed to collect adaptations to strong selection pressure. Almost all of the identified adaptations (97.9% of the p-adaptations and 99.8% of the e-adaptations) had the absolute values of the posterior means of the selection parameters greater than 0.2.

### Coadaptation and enrichment analysis

To examine the pattern of coadaptation, we listed the traits and gene expressions that adapted along each edge of the admixture graph. The size of coadaptations was measured as the sum of each row of the significance matrix. We functionally characterized the large-scale coadaptations by enrichment analysis of STRING (Szklarczyk et al. 2023). We also performed enrichment analysis of the causal genes to characterize the molecular background of the QTLs. We used the R package rbioapi (Rezwani et al. 2022) to access the STRING database.

## Results

### Range expansion, migration, and introgression from relicts

**Fig. 1.**
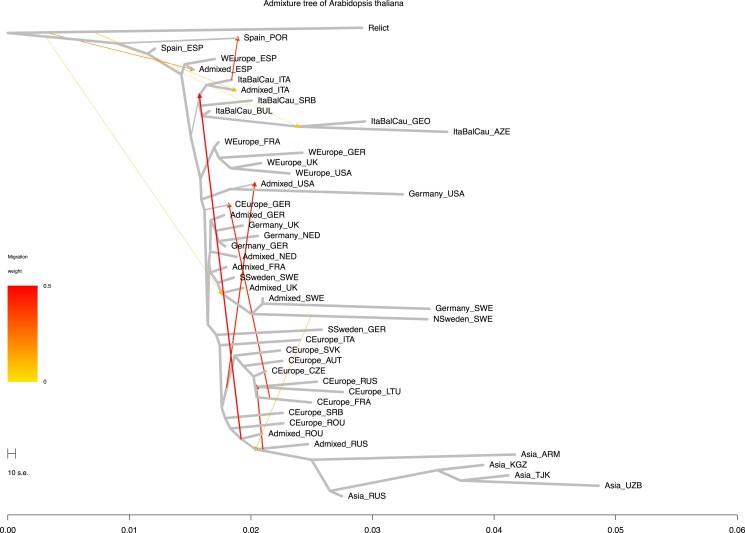
The admixture graph of *Arabidopsis thaliana*. The history of population divergence and admixture was estimated by TreeMix, setting relict as an outgroup. The admixture graph was assumed to include 10 admixture events (see Methods and Materials). The horizontal scale represents the factor that reflects the amount of genetic drift. The horizontal length of each edge can be interpreted as the ratio of the number of generations passed along the edge to the effective population size. The 3 admixture events from the edge leading to the relict to the edges leading to the nonrelict populations were interpreted here as introgression. The terminal nodes are defined by the genetic clusters of ADMIXTURE (*k* = 9, The 1001 Genomes Consortium 2016) subdivided by country (see Materials and Methods 2.2). The genetic clusters are labeled as Asia, Central Europe (CEurope), Germany, Italy–Balkan–Caucasus (ItaBalCau), Northern Sweden (NSweden), Southern Sweden (SSweden), Spain, and Western Europe (WEurope). The countries are labeled as Armenia (ARM), Austria (AUT), Azerbaijan (AZE), Bulgaria (BUL), Czech Republic (CZE), Spain (ESP), France (FRA), Georgia (GEO), Germany (GER), Italy (ITA), Kyrgyzstan (KGZ), Lithuania (LTU), Netherlands (NED), Portugal (POR), Romania (ROU), Russian Federation (RUS), Serbia (SRB), Switzerland (SUI), Slovakia (SVK), Sweden (SWE), Tajikistan (TJK), United Kingdom of Great Britain and Northern Ireland (UK), the United States of America (USA), and Uzbekistan (UZB).

Figure 1 shows the history of population splitting and admixture by TreeMix. The set of Relicts was specified as an outgroup (Materials and Methods). As the global pattern of nonmigrant edges was qualitatively unchanged across different values of *m*, the prespecified number of admixture events (Supplementary Fig. 3), we adopted the m=10 admixture graph for the subsequent analyses of p-adaptations and e-adaptations (see Methods and Materials for more detailed explanation). The horizontal length of each edge can be interpreted as the ratio of the number of generations passed along the edge to the effective population size. Although we cannot separate the 2 factors at this stage, visual inspection of the graph suggests that the effective population size varies between lineages. For example, the lineages from the ancestral node of the Central European and Asian populations to the Asian groups are much longer than the lineages from this ancestral node to the Central European groups. Since both of the lineages experienced the same number of generations, the difference in length of the lineages implies that the lineage leading to the Asian groups had smaller effective population size. Similarly, the lineage of the Italy–Balkan–Caucasus genetic cluster leading to Georgia and Azerbaijan and the lineage of the German genetic cluster leading to the United States were suggested to have small effective population sizes. The 3 admixture events from the edge leading to the relict to the edges leading to the nonrelict populations were interpreted here as introgression, allowing us to assess how these events increased genetic variation in populations and contributed to adaptation to the local environment (Lee et al. 2017).

The population in Iberia was consistently clustered with relicts in the admixture graphs with different settings for the number of admixtures. The other populations were mostly grouped in the genetic clusters by ADMIXTURE: Italy–Balkan–Caucasus, Western Europe, Germany and Sweden, Central Europe, and the populations in Asia derived from Central Europe. Their vertically straight arrangement indicates their spread in a short period of time, reflecting the history of human activity dispersal (François et al. 2008, Lee et al. 2017). The long edges toward Georgia and Azerbaijan, the United States, Sweden, and Asian countries may reflect population bottlenecks in migrations.

The colonization history within the same genetic clusters can be interpreted as the migration into an open niche. For example, the splitting between the United States and the United Kingdom in the Western European cluster corresponds to the migration from Great Britain to the American continent. Similarly, the edge to the United States in the German genetic cluster corresponds to the migration from the European continent. In addition, the United States received the migration from Central Europe. This picture of multiple sources of introduction from across Eurasia (Shirsekar et al. 2021) could not be captured if the countries were treated as the unit of the population (Supplementary Fig. 4). The order of the splits in Supplementary Fig. 4 was partly inconsistent with the order observed in Fig. 1. This may be due to the uncertainty in the estimation and of misspecification of the population units.

### Identified history of p-adaptations and e-adaptations

Out of 248 phenotypes that had identified QTLs with *P*-values less than $10^{-6}$, 220 had more than 1 QTLs (Supplementary Fig. 5). The mean number of QTLs was 362.04, and the maximum was 11,518. Out of 2,879 gene expressions that had identified eQTLs with *P*-values less than $10^{-6}$, 1,438 had more than 1 eQTLs. The mean number of eQTLs was 7.61, and the maximum was 1,159.

By focusing on the phenotypes and gene expressions that have QTLs/eQTLs of biallelic SNPs and are measured in total for at least 3 individuals in each of the 46 populations, we estimated the selection on 174 phenotypes and 1,829 gene expressions. The traits and genes with many QTLs and eQTLs tended to have undergone multiple times p- and e-adaptations in the admixture graph (Supplementary Fig. 6). This may be partly because the power of PolyGraph to detect selection increases with the number of QTLs and eQTLs. However, the enrichment analysis of genes with identified e-adaptations listed many specific responses to biotic and abiotic stresses such as response to light stimulus, photosynthesis, response to cold, response to hypoxia, response to nitrogen compound, response to bacteria, and immune system process (Supplementary Table 1). On the other hand, the enrichment analysis of genes with no identified e-adaptations listed few broader GO terms, response to organic substance, response to stimulus, and response to chemical, with function of catalytic activity and binding (Supplementary Table 2).

We identified 650 p-adaptations and 3,925 gene e-adaptations (e-adaptations) in the population history [false discovery rate (FDR) = 0.05; Supplementary Table 3]. All the α values (selection parameters) and their *z*-values are found in Supplementary Data 1–4. The identified p-adaptations and e-adaptations at each edge of the admixture graph and the enrichment analysis can be found in Supplementary Data 5 and 6.

### Four lineages with large-scale trait and adaptations and gene e-adaptations

**Fig. 2.**
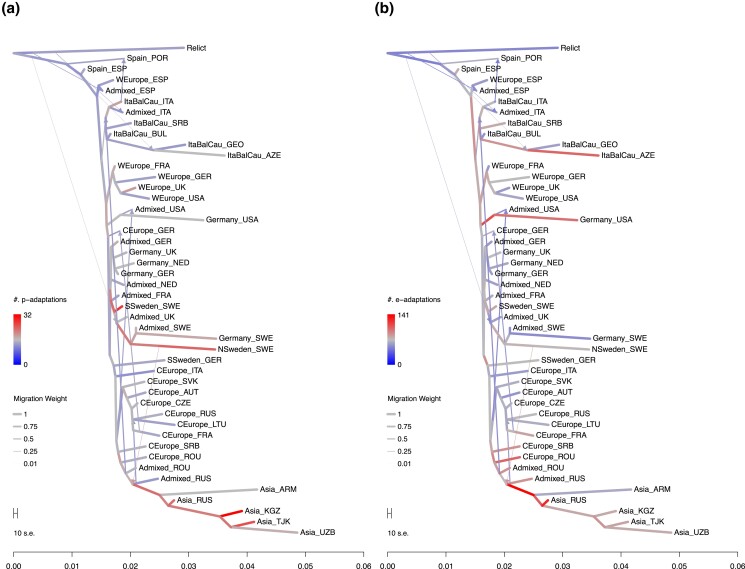
The number of p-adaptations and e-adaptations along the edges of the admixture graph. a) The number of p-adaptations, b) The number of e-adaptations. The edge width represents the migration weight. The terminal nodes are defined by the genetic clusters of ADMIXTURE (*k* = 9, The 1001 Genomes Consortium 2016) subdivided by country (see Materials and Methods 2.2).

**Fig. 3.**
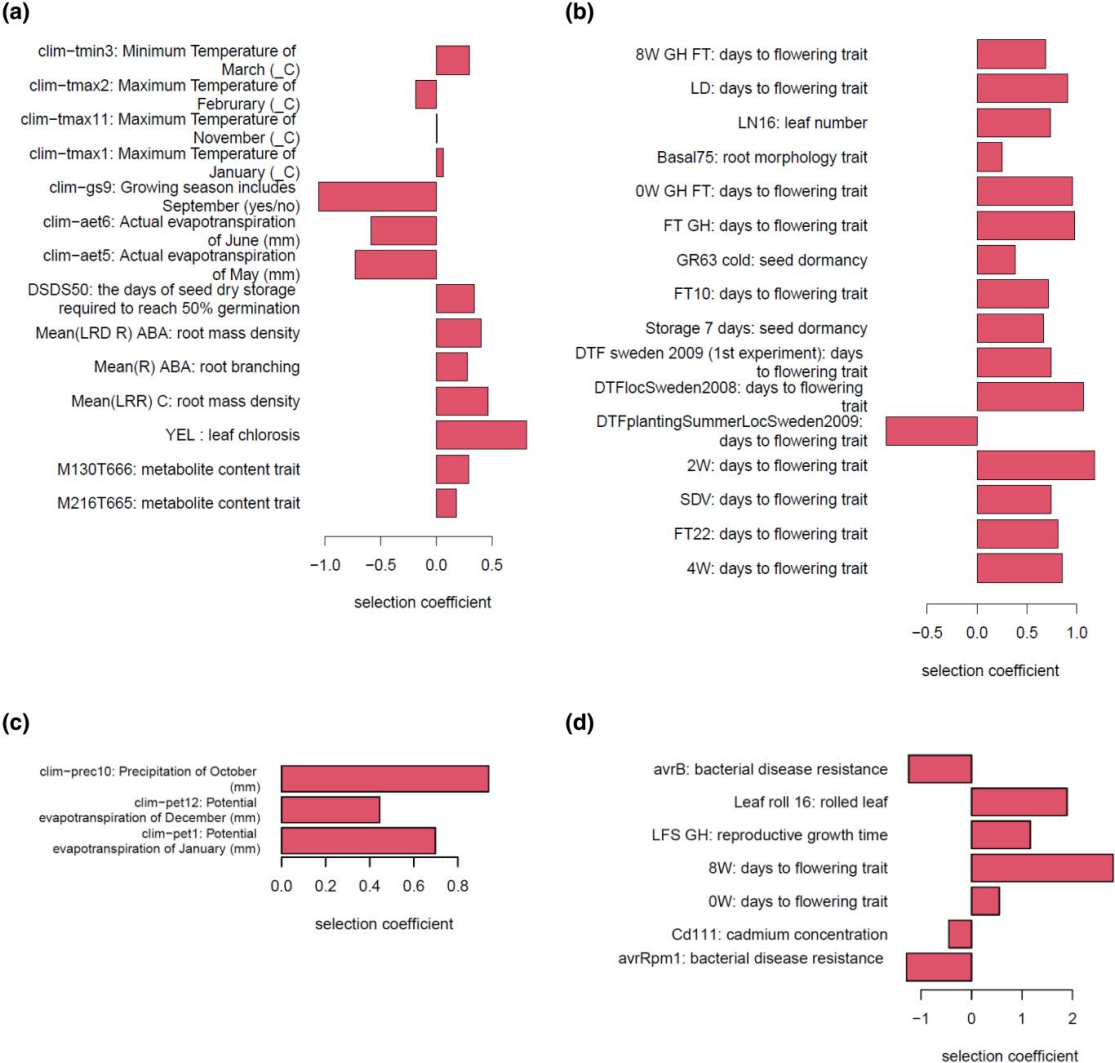
The p-adaptations along the 4 lineages. Mean selection coefficients within the lineages are shown. a) diverged from Central Europe and led to the Asian admixture group: Kyrgyzstan, Tajikistan, and Russia, b) led to Sweden, c) in the Italy–Balkan–Caucasus group led east to Azerbaijan, and d) led to the United States.

**Fig. 4.**
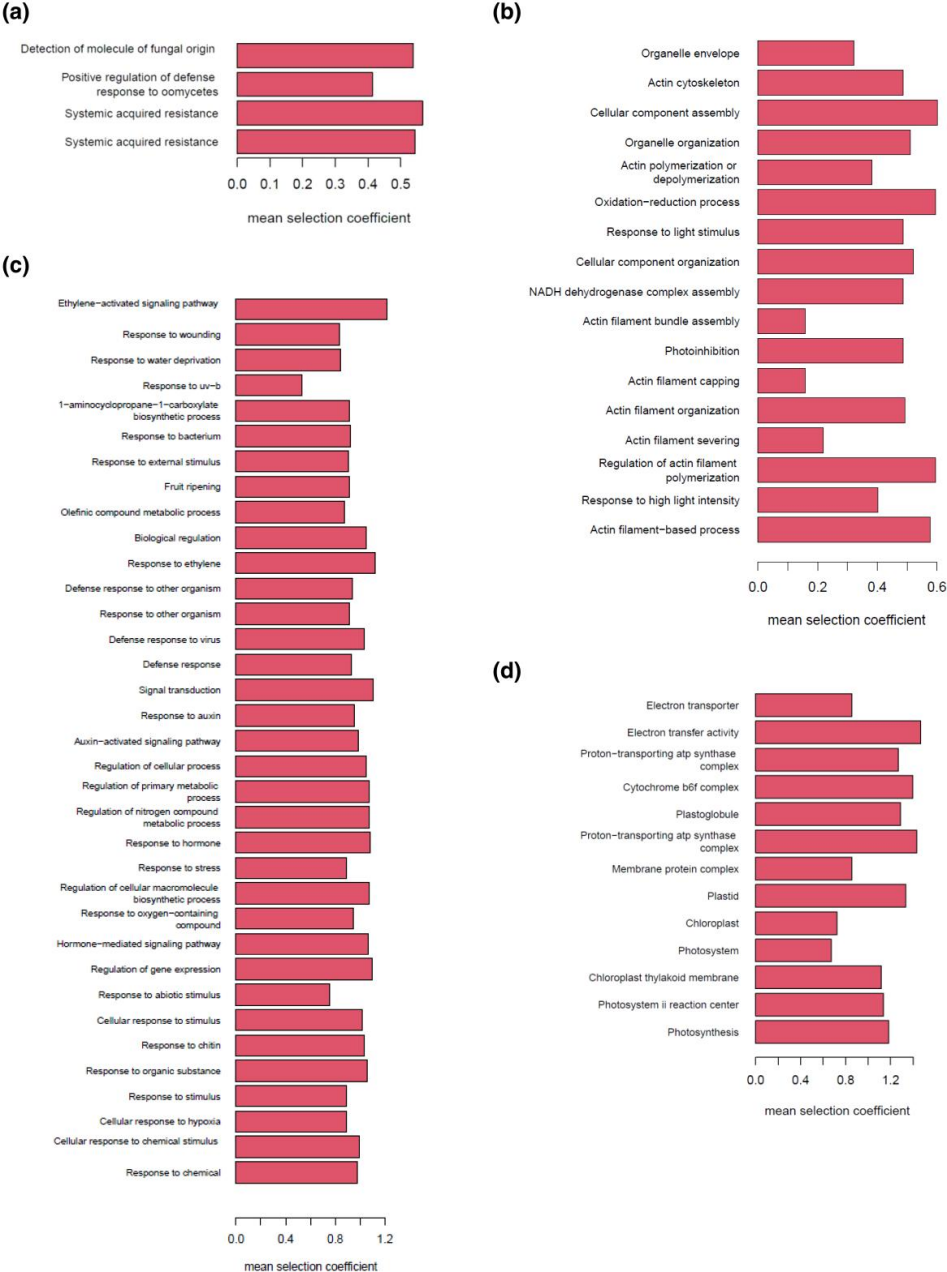
The enrichment analysis of e-adaptations along the 4 lineages. Mean selection coefficients within the lineages are shown. a) diverged from Central Europe and led to the Asian admixture group: Kyrgyzstan, Tajikistan, and Russia, b) led to Sweden, c) in the Italy–Balkan–Caucasus group led east to Azerbaijan, and d) led to the United States.

Among these, large-scale p-adaptations occurred along the 2 lineages (Fig. 2a). One is diverged from Central Europe and leads to the Asian admixture group, Central Asia (Kyrgyzstan and Tajikistan), and southern Siberia (Russia). The other is the lineage toward Sweden. Three lineages (Fig. 2b) have also undergone large-scale e-adaptation. One is the above lineage toward the Asian admixture group. The others are the lineage from Germany to the United States and the lineage in the Italy–Balkan–Caucasus group east to Azerbaijan. In particular, the eQTLs of more than a hundred genes changed their allele frequencies at each lineage edge in response to the environmental stress (Supplementary Table 4; Fig. 7). In this paper, we focus on these 4 lineages. To characterize the adaptations along the lineage, we collected the traits and gene expressions that were adapted on more than half of the edges. Figure 3 shows the mean selection coefficient of the identified p-adaptations, and Fig. 4 shows the mean selection coefficient of the identified e-adaptations.

### Eastward migration into Central Asia and southern Siberia, Russia

The *A. thaliana* population underwent large-scale adaptation of traits and gene expression along the 7 edges leading into Kyrgyzstan, Tajikistan, and Russia (Fig. 2; Supplementary Fig. 7; Supplementary Data 5 and 6). The Russian samples in the Asian admixture group were collected in southern Siberia (Supplementary Fig. 8).

Figure 3a starts with the stress of the low temperature in late autumn and late winter. The stronger the selection for shorter growing season, the lower the evapotranspiration in early summer. Remarkable p-adaptations are seed dormancy, leaf chlorosis, root mass density, and root branching in response to phytohormonal signaling pathways (Fig. 3a; Supplementary Table 4). Cold winters and low precipitation characterize the climate of the sampling sites in southern Siberia (Supplementary Fig. 9). In response to the very cold mean temperature (−21°C in January), the population prolonged seed dormancy and delayed germination (Bewley 1997; Alonso-Blanco et al. 2003). The short growing season required efficient growth, as summer annuals germinate in spring and seed in late summer (Footitt et al. 2013; Huang et al. 2014). The climates of Kyrgyzstan and Tajikistan are very similar, characterized by cold winters (−6.4 to −9.3°C from December to February) and high rainfall in spring (314 to 406 mm from March to May), followed by hot and dry summers (28 to 31°C and 39 to 41% humidity in July and August) (Supplementary Fig. 7). Plants in the populations reduced evapotranspiration in May and June. They adapted to the dry summer by expanding the root system architecture (RSA): the rate of proliferation and differentiation, the direction of root growth, and the formation of lateral roots (Ristova et al. 2018). It also enabled rapid growth in a short growing season by efficiently taking up the water and nutrients needed for growth.

Enrichment analysis of e-adaptations identified PR1, PNP-A, AED1, and RLP23 whose functions are the systemic acquired resistance in response to oomycetes and detection of molecules of fungal origin (Fig. 4a; Supplementary Table 4; Supplementary Data 7). The mean maximum temperature near the sampling sites in July is 24°C in Novosibirsk in the southern Siberia and 29 to 31°C in Kyrgyzstan and Tajikistan (Supplementary Fig. 9). Fungal and bacterial growth rates had optimum temperatures around 25–30°C (Pietikäinen et al. 2005). Growth rates decrease at either higher or lower temperatures. The decrease is more drastic for bacteria at higher temperatures, while it is more drastic for fungi at lower temperatures. As a result, fungi are better adapted to low-temperature conditions than bacteria (Pietikäinen et al. 2005). Oomycetes become most aggressive against lucerne when a cool, wet spring is followed by an early, warm, dry summer (Abbas et al 2022). The identified p-adaptation of leaf chlorosis is the penetration resistance in response to microbial invasion (Johansson et al. 2014). It was also an adaptation to the short growing season. By closing the path to the leaves, more nutrients are directed to the seeds, and seed maturation is accelerated as the leaves turn yellow during the reproductive process (Huang et al. 2014).

### Migration northward to Sweden

In Sweden, *A. thaliana* has adapted to cold winters by delaying flowering time (Fig. 3b; Supplementary Table 5; Supplementary Data 5 and 7), as was observed in Ågren and Schemske (2012). It behaves as winter annual, germinating in autumn, overwintering as a vegetative rosette, and flowering in spring or summer (Li et al 2010; Ågren and Schemske 2012; Footitt et al. 2013). The Swedish ecotype is exposed to subfreezing mean monthly minimum temperatures for several winter months (Supplementary Fig. 9) and has a higher freezing tolerance. The other major adaptation is to acquire sufficient carbon to support reproduction during the growing season. The Swedish ecotype has extended its life cycle with earlier seedling establishment (early September) and late seed maturation (late June). As a result, it experiences similar temperatures to the Italian ecotype at corresponding developmental stages (Demmig-Adams et al. 2022).

Enrichment analysis of e-adapted genes identified ATH13, DIS1, FAD6, FTSH8, KAC2, NDA1, SIGE, VLN2, and VLN3 that have the role of actin filament in response to light stimulus (Fig. 4b; Supplementary Table 5; Supplementary Data 6 and 7). Actin filaments play an essential role in organelle movement in plants (Kadota et al. 2009). Chloroplasts move in response to external stimuli, in particular light. Low light induces a chloroplast accumulation response so that light is captured efficiently for photosynthesis. Strong light induces an avoidance response to avoid light damage. Overexpression of F-actin genes and p-adaptation of an increased number of leaves allowed efficient photosynthetic activity in response to the low irradiance.

Although the Central Asia and Sweden both have cold winters, *A. thaliana* has adopted contrasting strategies. The average minimum temperature is lower in Bishkek, Kyrgyzstan (−9.3°C) and Dushanbe, Tajikistan (−8.2°C), than that of Stockholm, Sweden (−3.9°C), where majority of samples were collected in these regions (https://en.climate-data.org/). Bishkek and Dushanbe are dry (39–45% humidity in July and August), while Stockholm is humid throughout the year (69–87% humidity) (Supplementary Fig. 9). Summer is cooler and shorter in Stockholm (maximum temperature is 21°C in July) than in Bishkek (29°C) and Dushanbe (31°C). Sunlight is stronger in Central Asia. These environmental conditions led to different adaptation strategies, summer annuals with rapid growth in Central Asia, and winter annuals with longer life cycles in Sweden.

### Migration to Azerbaijan

A large-scale e-adaptation was identified along the lineage in the Italy–Balkan–Caucasus group eastward to Azerbaijan (Fig. 2; Supplementary Fig. 7). The p-adaptations imply the climatic change experienced by the population on the way to Azerbaijan (Fig. 3c; Supplementary Table 6; Supplementary Data 5). The climate of Azerbaijan is very diverse, with 9 of the world's 11 climate zones, including semiarid, temperate, continental, and tundra zones. This results in significant variations in annual temperature and precipitation across the country (https://climateknowledgeportal.worldbank.org/).

Enrichment analysis identified the plant hormone signaling pathways and enhanced responses to external stimuli (ACS6, ERF017, ERF016, PILS7, CAF1b, ECS1, ERF13, GH3.3, HLS1, IAA13, IAA19, IAA5, NHL3, PDF1.3, PROPEP3, RRTF1, RTM3, TIP, WRKY40, and WRKY46) (Fig. 4c; Supplementary Table 6; Supplementary Data 6). Plant hormones regulate various physiological processes, including plant defense. Among them, jasmonate (JA) and salicylic acid (SA) are important defense-related phytohormones. Ethylene (ET), abscisic acid (ABA), auxin, gibberellins (GAs), cytokinins (CKs), and brassinosteroids (BRs) are also involved in defense responses (Kazan and Manners 2009, Berens et al. 2017).

These adaptations reflect the diversity of insects and microbes generated by the local climate. The sample was taken from the south-eastern corner (Supplementary Fig. 10). Lankaran, the sampling site in Azerbaijan, has a hot-summer Mediterranean climate (Csa) and is the wettest province in the country: the mean temperature in July exceeds 26°C, the mean annual precipitation is 1200 mm, and the mean number of rainy days is 110 days; 42% of the annual precipitation and 32% of the rainy days occur between September and November (35 days, 540 mm; Supplementary Fig. 9). Based on the latitudinal proximity to the Central Italy and similar annual patterns of temperature, precipitation, daylight hours, and humidity (figure not shown), the germination and flowering time in Lenkaran are expected to be similar to those in Italy from October to December and February to April (Ågren and Schemske 2012). The population in Lenkaran would be affected by high rainfall before germination and during germination and growth of seedlings. The plant fungal pathogen *Fusarium oxysporum* is the causal agent of root rot or wilt diseases in several plant species (reviewed by Berrocal-Lobo and Molina 2008). Affected plants (hosts) are mostly from the tropical and subtropical areas, probably because wilt symptoms are more pronounced at elevated temperatures and humidity (Acharya et al. 2011).

### Migration of German population into the United States

The sample from the United States was mainly of Germany (Supplementary Fig. 11). The adapted traits of the accompanying weeds were days to flowering, bacterial disease resistance, leaf cadmium concentrations, and rolled leaf (Fig. 3d; Supplementary Table 7; Supplementary Data 5). The selection for a reduction in leaf cadmium concentrations indicated that the plants were exposed to heavy metals. The enrichment analysis of the QTL-coding genes of the cadmium concentrations in leaves (Cd111) indicated that P-type ATPase, transmembrane transporters reduced their activities (Supplementary Table 8; Supplementary Data 5 and 7). Cadmium is mainly released from nickel–cadmium batteries, fossil fuel combustion, coating and plating, and cement production (Ruchi Bharti and Sharma 2022). In fact, the samples were mainly collected from the coastal areas of Lake Michigan and near New York City (Supplementary Fig. 11). The state of Michigan produces cement and has a mining industry.

The heavy metal stress selected the alleles with lower germination rate, lower leaf number, slower growth and flowering, and rolled leaves, providing a chance for succession in an improved environment (Li et al. 2005). Notably, alleles with weak resistance to bacteria were selected. This is probably because the heavy metals affected not only the plant individuals but also plant pathogens. As a result, the plants had a reduced risk of bacterial infection. As disease resistance comes at some cost (van Hulten et al. 2006), alleles with lower resistance were selected in the absence of infection risk.

Enrichment analysis of the e-adapted genes identified the selection of alleles with higher expression of the photosynthesis-related genes (AT2G12905, ATPA, ATPH, MATK, ORF31, PB, PETD, PETG, PSAB, PSAJ, PSBE, PSBF, PSBI, PSBJ, PSBK, PSBL, PSBT, RBCL, SIGE, and YCF10) (Fig. 4d; Supplementary Table 7; Supplementary Data 6). *Arabidopsis* is a C3 plant that performs C3-type photosynthesis, as do algae such as *Chlorella* and many plants such as rice, wheat, soybean, rapeseed, and spinach. Unlike C4 plants, photosynthesis in C3 plants is not carried out by the division of labor between leaf pith cells and vascular sheath cells. They are therefore less able to capture CO_2_ under conditions that tend to close their stomata, such as high temperatures and drought. Photosynthesis is less efficient in climates that are hostile to plants, such as high temperatures, drought, low CO_2_, and low-nitrogen soils (Brown 1978, Waller and Lewis 1979). In addition, the heavy metals weaken the photosynthetic activities. Populations have adapted to this activity-depressing pressure by selecting for alleles with higher expression of photosynthetic-related genes.

### Selection on the haplotypes

Although *A. thaliana* is a selfing weed, linkage disequilibrium decays rapidly, within 50 kb (Nordborg et al. 2005). Notably, however, we further identified selection on the haplotypes of the adaptive QTLs and eQTLs. Selection was particularly strong along the migration lineage from Germany to the United States. Here we describe the selection on the haplotypes of the QTLs and eQTLs, focusing on the observation of this lineage.

Each of the QTLs and eQTLs had 2 alleles “0” and “1.” Allele “1” was defined as an H-allele (higher value/higher expression), and the allele “0” was defined as L-allele (lower value/lower expression), if the effect size of the QTL/eQTL was positive. If the effect size was negative, the allele “1” was defined as L-allele and the allele “0” was defined as H-allele. If a trait/gene expression has *n* QTLs/eQTLs, the number of haplotypes becomes $2^{n}$. PolyGraph estimates the unidirectional selection for increasing or decreasing the frequencies of the H-alleles of the QTLs/eQTLs in the populations. To see the unidirectional selection on the haplotype of each individual, we examined a characteristic of the haplotypes, that is, the proportions of the H-alleles among the QTLs/eQTLs. We counted the proportions of H-alleles for each of the identified traits and gene expressions for each individual. As above, the enrichment analysis of e-adaptations identified 20 photosynthesis-related genes, AT2G12905, ATPA, ATPH, MATK, ORF31, PB, PETD, PETG, PSAB, PSAJ, PSBE, PSBF, PSBI, PSBJ, PSBK, PSBL, PSBT, RBCL, SIGE, and YCF10. They had a total of 108 eQTLs, widely distributed on chromosomes 1 and 2. Of these, 51 significantly changed allele frequencies (FDR = 0.05) along this lineage.

**Fig. 5.**
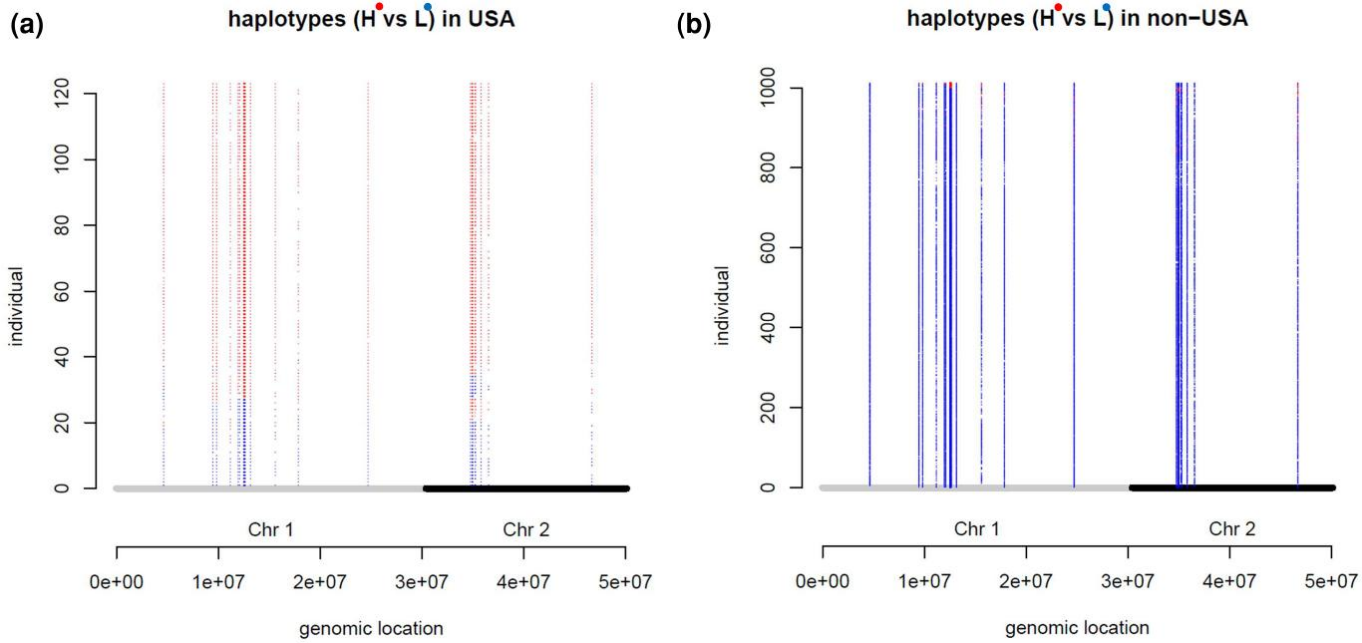
Haplotypes of the photosynthesis-related eQTLs, whose allele frequencies changed significantly (FDR = 0.05) along the lineage to the United States. Each horizontal line of dots represents an individual's haplotype. a) Individuals from the United States sample and b) individuals from the non-US sample. The photosynthesis-related genes that were identified in the enrichment analysis of e-adaptations along the lineage to the United States. Each eQTL has an H-allele (higher expression) and an L-allele (lower expression).

Out of 123 individuals in the US sample, 84 individuals (68%) had H-alleles at all eQTLs (Fig. 5a). We call this haplotype as a full haplotype. These 84 individuals were all from the German admixture group (Supplementary Fig. 12). In sharp contrast, in the sample from outside the United States, 834 out of 1,032 individuals (82%) had L-alleles (lower expression) at all eQTLs (Fig. 5b). However, in the German sample, no individual had more than a few H-alleles. No individual in the UK sample had the full haplotype either, although a few individuals had H-alleles at about 80% of the eQTLs.

**Fig. 6.**
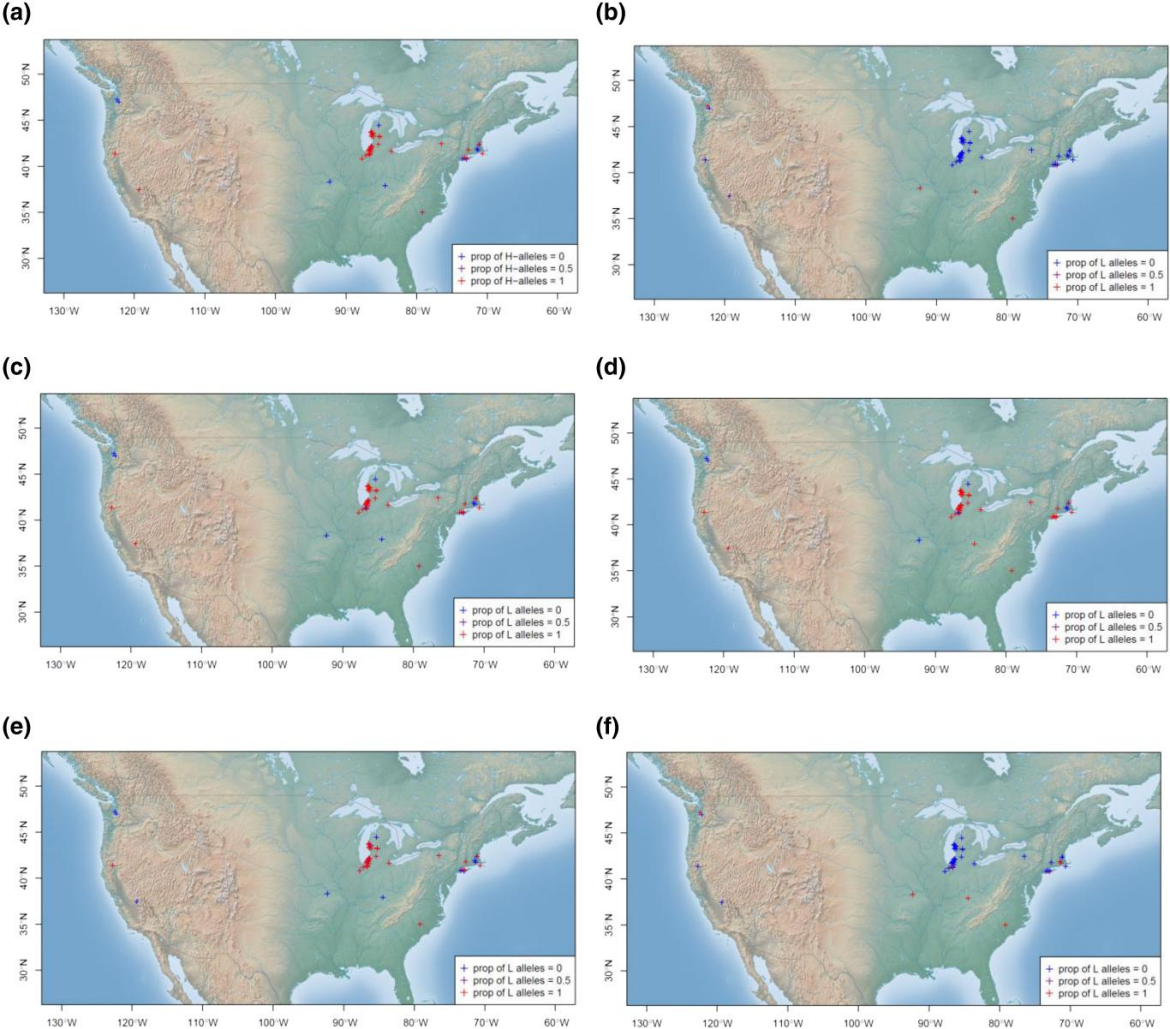
The geographical map of the United States sample. The haplotypes of the sampled individuals are characterized by the proportions of the H-alleles. a) eQTLs of photosynthesis-related genes identified by the enrichment analysis of e-adaptations, b) QTLs of cadmium concentration in leaves, c) QTLs of reproductive growth time (number of days between germination and senescence of the last flower), d) QTLs of the trait days to flowering (number of days required for the bolt height to reach 5 cm), e) QTLs of rolled leaf, and f) QTLs of bacterial resistance.

The haplotypes of photosynthesis-related eQTLs received a strong selection for increasing H-alleles in the coastal area of Lake Michigan, the urban areas near New York City, and the arid area of California (Fig. 6a). At the same time, these areas showed strong selection on alleles of numerous QTLs for decreasing heavy metal uptake activity (Fig. 6b), increasing the length of growing season (Fig. 6c and d), increasing rolled leaf (Fig. 6e), and decreasing bacterial resistance activity (Fig. 6f).

## Discussion

We identified 650 p-adaptations and 3,925 e-adaptations in the population history of *A. thaliana* (Supplementary Table 3; Supplementary Data 5 and 6). In this paper, we focused on the 4 lineages that experienced a large-scale p-adaptations and e-adaptations and characterized the adaptations (Supplementary Data 7). In these lineages, the relative fitness of the alleles at many QTLs and eQTLs changed. The populations also seemed to optimize the site frequency spectrum of these loci to fit to the environment. In the eastward migration to Central Asia and southern Siberia, the extremely cold winter and short summer prolonged seed dormancy and expanded the RSA. In the northward migration to Scandinavia, low temperatures prolonged the growing season, and low light intensity required the increased chloroplast activity. The subtropical and humid environment of south-eastern Azerbaijan enhanced phytohormone signaling pathways in response to the biotic and abiotic stresses.

The migrated *Arabidopsis* accessions adapted to the heavy metal exposure and arid environment of the United States by selecting the eQTL alleles that increase photosynthetic activity, the QTLs that reduce the growth rate, and those that reduce the weight of bacterial resistance. Figure 6 suggests that the population had completed the full set of adaptations by the time it reached the east coast and that the adaptive haplotypes spread westward. However, these haplotypes were not present in the German sample. Instead, these haplotypes may have been introduced through migration events (Supplementary Fig. 12). An alternative tempting scenario is that an environmental change in Germany wiped out the haplotypes, but we could find no evidence for it. With the current approach, it is still difficult to determine patterns and signatures of polygenic adaptation at the molecular level. Focused study of the North American *A. thaliana* population revealed that multiple introductions into a nonnative range have allowed a rapid adaptation of the colonizing species by increasing haplotypic diversity through admixture (Shirsekar et al. 2021). It is interesting to scrutinize the large-scale p-adaptations and e-adaptations observed in our analysis with reference to result from the focused study with enhanced fine-scale population genomic data.

In TreeMix analysis, we tentatively set the number of admixture events, *m*, to be 10. OptM suggested m=1 among the candidate values of m=1,…,15, but the composite likelihood did not reach the plateau but continued to increase linearly. The variance explained was 93%, far below the value of 99.8% suggested by the authors of TreeMix (Supplementary Fig. 13). Since the standard deviation measured sensitivity of the maximum composite values against initial values, we also conducted OptM by block resampling of genomic regions consisting of 300 SNPs to account for the uncertainty due to sampling of SNPs. Although *A. thaliana* is selfing, the linkage disequilibrium decays within 50 kb. Since the genome size of *A. thaliana* is $1.35\times 10^{8}$ and noting that our TreeMix is based on the 37,718 neutral SNPs, the average block size is $300\times (1.35\times 10^{8}/37,718)∼1\;(\mathrm{Mbp}).$ Therefore, blocks can be assumed independent among others. The standard deviation of the composite likelihoods became much larger, but the pattern of the composite likelihood was qualitatively unchanged (Supplementary Fig. 14). The value of m=1 was suggested, because the composite likelihood was improved by incorporating a single admixture event, and the slope of the linear increment was lower compared with the difference between the case of m=1 and the case of m=0. Although we set the maximum value of *m* for practical computational reasons, the output of OptM seemed to imply an insufficient range of *m* for comparison. On the other hand, the global pattern of nonmigrant edges was qualitatively unchanged across different values of *m* (Supplementary Figs. 3 and 15). As a compromise between sufficient fit to the data and the computational feasibility, we decided to use the m=10 admixture graph for the subsequent analyses in this paper. The sensitivity of the downstream PolyGraph and enrichment analysis to the prespecified number of the admixture edges is left for future study.

**Fig. 7.**
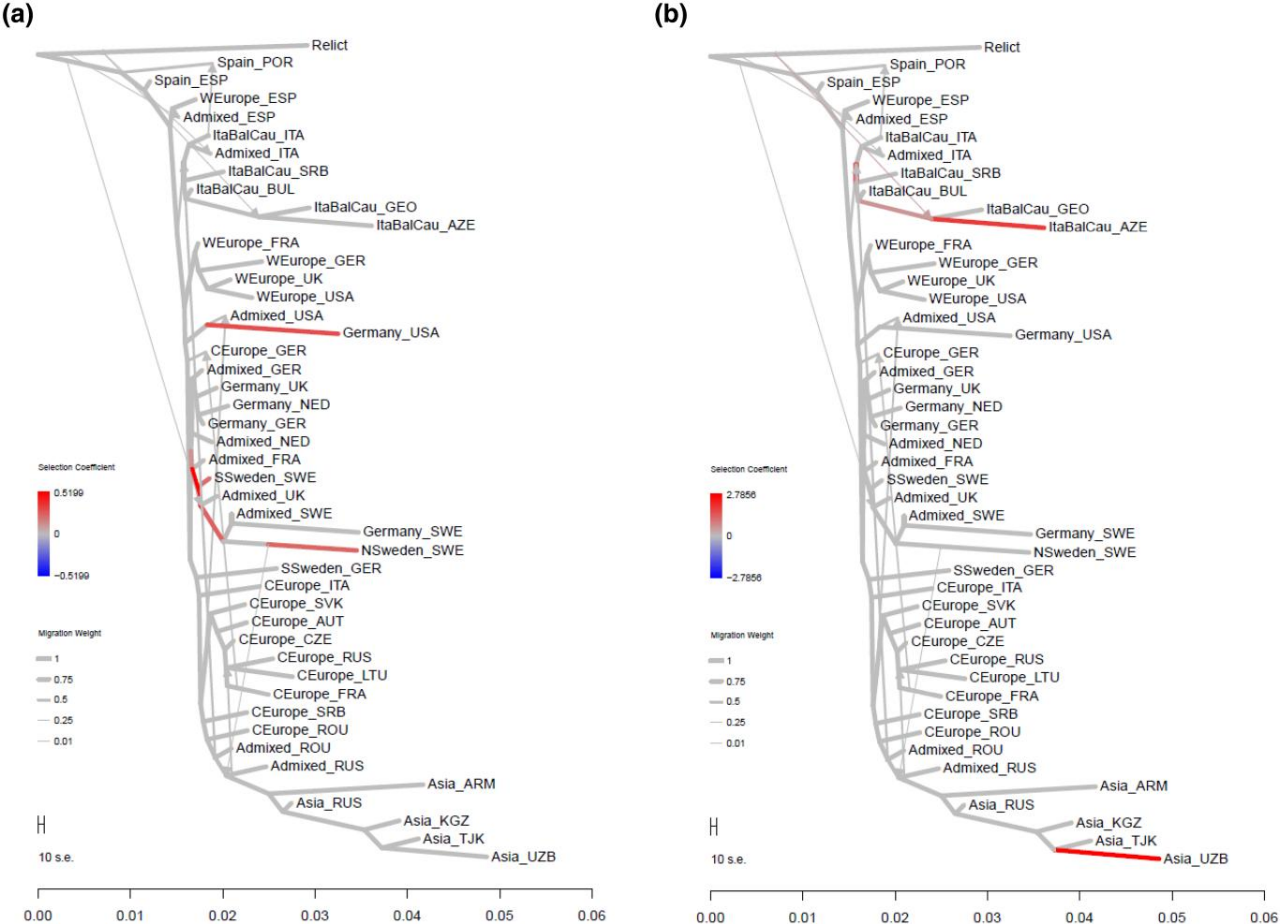
Example cases of convergent/parallel p-adaptation and e-adaptation. a) The p-adaptation of the trait pid119: the number of days required to reach flowering stage from a predetermined time point (e.g. the date of seed sowing, seedling transplant, or seedling emergence), and b) the e-adaptation of WRKY40 (AT1G80840), the pathogen-induced transcription factor.

The pdf files in Supplementary Data 1 and 2 contain the identified p-adaptations of each trait and the e-adaptation of each gene expression on the admixture graph. Visual inspection of these p-adaptations and e-adaptations revealed many likely convergent/parallel adaptations. For example, the alleles that increased the time from seedling to flowering were selected during the migration to the United States and during the migration to Northern Sweden (Fig. 7a). Alleles that increased the expression of WRKY40, a pathogen-induced transcription factor, were selected during the migration to Azerbaijan and during the migration to Uzbekistan (Fig. 7b). In order to characterize the convergent/parallel adaptations, we had collected p- and e-adaptations along the edge pairs of the admixture graph. However, we were not prepared to report systematically on the convergent/parallel adaptation. This is partly because, as can be seen in Fig. 7, some edge pairs are included in a series of successive edges, and some represent adaptation during the migration of different genetic clusters into the same country. The statistical analysis of convergent/parallel adaptation is left for the future study.

The seasonal timing of seed germination determines a plant's realized environmental niche. To characterize the genetic basis and climatic associations of natural variation in seed chilling responses, Martínez-Berdeja et al. (2020) conducted GWAS for 559 accessions from across a wide climate range. They found that the alleles of the DELAY OF GERMINATION 1 (DOG1) of Spanish lowland ecotypes have a DNA sequence deletion that produces an amino acid loss, possibly causing the reduced function. Using their data, Exposito-Alonso (2020) synthesized the seasonal timing adaptation into 4 categories: Mediterranean rapid cyclers, facultative winter cyclers, weedy summer/spring cyclers, and strict Scandinavian winter cyclers. To see how this gene was included in our analysis, we searched for the traits that had QTLs on the gene and found that 15 traits of days to flowering had QTLs on DOG1 (AT5G45830). Although we did not list the genes causing the polymorphism of the traits, these QTLs contributed to the identification of the p-adaptation of flowering times. No eQTLs were identified for this gene. However, its expression level was reduced in the Mediterranean countries, Spain, Portugal, and France (Supplementary Fig. 16). The selection on this DOG1 allele of amino acid loss was identified as a p-adaptation in our analysis but may also be an e-adaptation that was not identified by its eQTLs. Statistical modeling incorporating this type of e-adaptation is left for the future study.

Enrichment analysis was useful to characterize the sets of e-adaptations in response to the local environments. It was also useful to characterize the p-adaptations. Alleles at the QTLs in the coding regions may affect the traits mainly by altering the physical and chemical properties of the relevant proteins, while those in the intergenic regions may affect the traits by altering the gene expression. In this paper, we did not fully analyze the latter type of QTLs to characterize the molecular mechanisms behind the p-adaptations, but the enrichment analysis of the QTL-accommodating genes could interpret the p-adaptations of cadmium concentration in leaves in the United States as the reduced activities of the transmembrane transporters.

It should be noted that the local environment may not necessarily select for the alleles of the key genes, as its changes may have deleterious effects on the pathways. For example, in the anthocyanin biosynthesis pathways, upstream genes evolved more slowly than the downstream genes (Rausher et al. 1999). Pleiotropy inhibits the spread of alleles in response to directional selection on a focal trait (Otto 2004). In regions with relatively low recombination, selected variants affect more neutral sites through linkage (Corbett-Detig et al. 2015). It should also be noted that comparing phenotypes and gene expressions between populations does not necessarily identify adaptation, as the observed phenotypes and gene expressions are the result of a balance between the environmental stresses and the genetic responses. Common environment experiments and reciprocal transplantation experiments can identify the genetic adaptation behind this countergradient variation (Levins 1969; Conover and Schultz 1995). The directional changes in the allele frequencies at the QTLs/eQTLs provide direct evidence of population adaptations.

Based on the database of QTLs (Togninalli et al. 2020) and eQTLs (Lan et al. 2021) and the population genomic data (The 1001 Genomes Consortium 2016), we constructed a database of p-adaptations and e-adaptations in the population history of *A. thaliana*, which were characterized with reference to the information on the local environments. It complements studies focusing on specific aspects of adaptation. Although traits without identified QTLs and genes without identified eQTLs were not included in the analysis, it provided us a clue for understanding the complex history of biological adaptation. The information on p-adaptation is directly interpretable. The information on e-adaptation may provide a less biased picture of physiological adaptation. Both have been essential in characterizing the adaptations in the 4 lineages. For example, in the northward migration to Scandinavia, the p-adaptations confirmed that the population adapted to the cold winter by extending the growing season. Importantly, the e-adaptations indicated a physiological adaptation to the low sunlight intensity in the northern area. By increasing the expression of actin filaments, the chloroplasts in the leaves were able to move smoothly in response to the sunlight. The current study could not integrate the p-adaptations and e-adaptations into a unified framework. The database of p-adaptations and e-adaptations, which are publicly available (Supplementary Data 1 and 2), may be useful for future studies to obtain a complete picture of the biological adaptations of *A. thaliana* throughout its population history.

## Supplementary Material

jkad218_Supplementary_Data

## Data Availability

The authors affirm that all data necessary for confirming the conclusions of the article are present within the article, figures, tables, Supplementary material, and Supplementary data. The following detailed information is available at https://zenodo.org/record/7903201: The screened data for this study (genotype, trait, environment, and gene expression), the estimated population history and selection parameters, and the scripts for the analysis The pdf files of Supplementary Figs. 1–16 The csv files of Supplementary Tables 1–8, including the list of input genes and preferred names for enrichment analysis Supplementary Data 1–7 containing the detailed information on the identified p-adaptations and gene e-adaptations with the result of the gene annotation and enrichment analysis Supplemental material available at G3 online.
