# Supplementary material for: Genetic adaptations in the population history of Arabidopsis thaliana
Source: G3 (Bethesda). 2023 Sep 25;13(12):jkad218. doi: 10.1093/g3journal/jkad218 (PMC10700115; doi:10.1093/g3journal/jkad218)
Supplement: jkad218_Supplementary_Data [file jkad218_supplementary_data.pdf]

Supplementary information for

Genetic adaptations in the population history of *Arabidopsis thaliana*

Hirohisa Kishino, Reiichiro Nakamichi, Shuichi Kitada

This pdf file include:
Supplementary Methods
Supplementary Figures S1–S16
Supplementary Tables S1–S8

The following detailed information is available at https://zenodo.org/record/7903201:

1. the screened data for this study (genotype, trait, environment and gene expression), the estimated population history and selection parameters, and the scripts for the analysis,
2. the pdf files of Supplementary Figures S1–S16,
3. the csv files of Supplementary Tables S1–S8, including the list of input genes and preferred names for enrichment analysis,
4. Supplementary Data S01–S07 containing the detailed information on the identified phenotypic adaptations (p-adaptations) and gene expression adaptations (e-adaptations) with the result of the gene annotation and enrichment analysis.

Table of Content

Supplementary Methods
S1. Filtering of polymorphic sites for TreeMix and PolyGraph
S2. Traits/gene expressions and their QTLs/eQTLs for PolyGraph

Figure S1 The sampling points of Arabidopsis thaliana
Figure S2 Multidimensional scaling of genotype data
Figure S3 The admixture graph estimated by TreeMix
Figure S4 The admixture graph of the countries with ten admixtures
Figure S5 The distributions of numbers of QTLs and eQTLs
Figure S6 The traits and genes with many QTLs and eQTLs tend to have undergone multiple times p- and e-adaptations
Figure S7 The numbers of p-adaptations and e-adaptations along the edges of the admixture graph
Figure S8 The assignment of the sample from Russia to the admixture groups
Figure S9 Climates of the cities representing the sampling locations from the four lineages with large scale adaptations
Figure S10 The sampling points in Azerbaijan
Figure S11 The assignment of the sample from the United States to the admixture groups
Figure S12 The proportions of the H-alleles at the photosynthesis-related eQTLs, whose allele frequencies changed significantly (FDR=0.05) along the lineage to the United States
Figure S13 The output produced by OptM
Figure S14 The output produced by OptM obtained by block resampling of genomic regions consisting of 300 SNPs
Figure S15 The admixture graph estimated by TreeMix, assuming the number of admixture edges, $m = 1$
Figure S16 Variable mean expression levels of DOG1 among countries

Table S1 Enrichment analysis of the genes with identified e-adaptations
Table S2 Enrichment analysis of the genes with no identified e-adaptations
Table S3 The numbers of p-adaptations and e-adaptations along each of the edges of the admixture graph
Table S4 p-adaptations and enrichment analysis of e-adaptations along the lineage to Central Asia and South Siberia, Russia
Table S5 p-adaptations and enrichment analysis of e-adaptations along the lineage to Sweden
Table S6 p-adaptations and enrichment analysis of e-adaptations along the lineage to Azerbaijan
Table S7 p-adaptations and enrichment analysis of e-adaptations along the lineage to the United States
Table S8 Enrichment analysis of the QTL-coding genes of the cadmium concentrations in leaves (Cd111)

Reference for Supplementary information

Data S01 The estimated α values (selection parameters) of p-adaptations at each edge of the admixture graph
Data S02 The estimated α values of e-adaptations at each edge of the admixture graph
Data S03 The Z values of the estimated α values of p-adaptations at each edge of the admixture graph
Data S04 The Z values of the estimated α values of e-adaptations at each edge of the admixture graph
Data S05 p-adaptations at each edge (selected traits, annotation and enrichment analysis of causal genes)
Data S06 e-adaptations at each edge (selected gene expressions, annotation and enrichment analysis of these genes)
Data S07 p-daptations and e-adaptations along the four lineages

Supplementary Methods

S1. Filtering of polymorphic sites for TreeMix and PolyGraph

The VCF data provided by 1001 Genomes (1001genomes_snp-short-indel_with_tair10_only_ACGTN.vcf.gz) contained 12,883,854 polymorphic sites. For population history inference by TreeMix, we filtered the sites for non-indels, biallelic, missing rate <1% and allele frequency > 1% (VCFtools option: --out SNPs --remove-indels --max-alleles 2 --min-alleles 2 --maf 0.01 --max-missing 0.99). Specifically, there were 11,458,975 non-indels, of which 10,707,430 were biallelic and 286,671 had a missing rate ≦ 1%. Finally, 37,718 SNPs remained with MAF≧ 1%. For the PolyGraph analysis, we used the subsets of these SNPs to contrast with QTLs and eQTLs. In total, 49,973 QTLs and 16,672 eQTLs were included in the AtMAD database. The 37,718 SNPs mentioned above contained 734 QTLs and 83 eQTLs. Since PolyGraph contrasts the allele frequencies of neutral SNPs with those of QTLs/eQTLs, we excluded them from the 37,718 SNPs, and used $36{,}984\ (= 37{,}718 - 734)$ SNPs for p-adaptation identification and $37{,}635\ (= 37{,}718 - 83)$ SNPs to identify e-adaptations.

S2. Traits/gene expressions and their QTLs/eQTLs for PolyGraph

The AtMAD database contained 49,973 QTLs and 16,672 eQTLs, which were subsets of the above 12,883,854 polymorphic sites. Among the 49,973 QTLs, 2 were non-biallelic. Of the 16,672 eQTLs, 2,933 were non-biallelic. PolyGraph contrasts the between-population variation in allele frequencies of the QTLs/eQTLs with that of the neutral SNPs. Unless some of the QTLs/eQTLs of a trait/gene expression were measured in at least one individual in each of the 46 populations, we had no way to apply PolyGraph to the trait/gene expression. If any of the QTLs/eQTLs of a trait/gene expression were measured in at least one individual in each of the 46 populations, we analyzed the trait/gene expression. As a result, we analyzed 174 traits out of 248 with identified QTLs and 1,829 gene expressions out of 2,879 with identified eQTLs. The number of QTLs and eQTLs that we analyzed was 23,880 and 8,618 respectively.

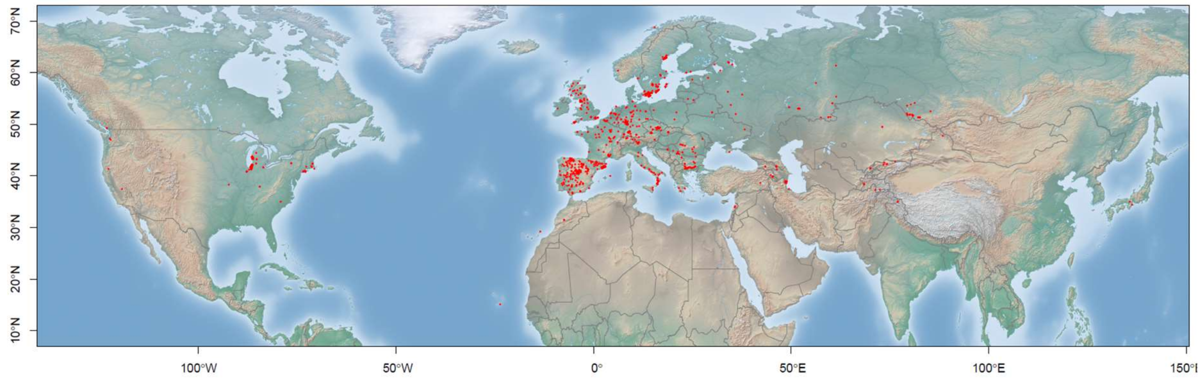

Figure S1 The sampling points of *Arabidopsis thaliana*. The geographic distribution of the samples recorded in AtMAD is represented by red points.

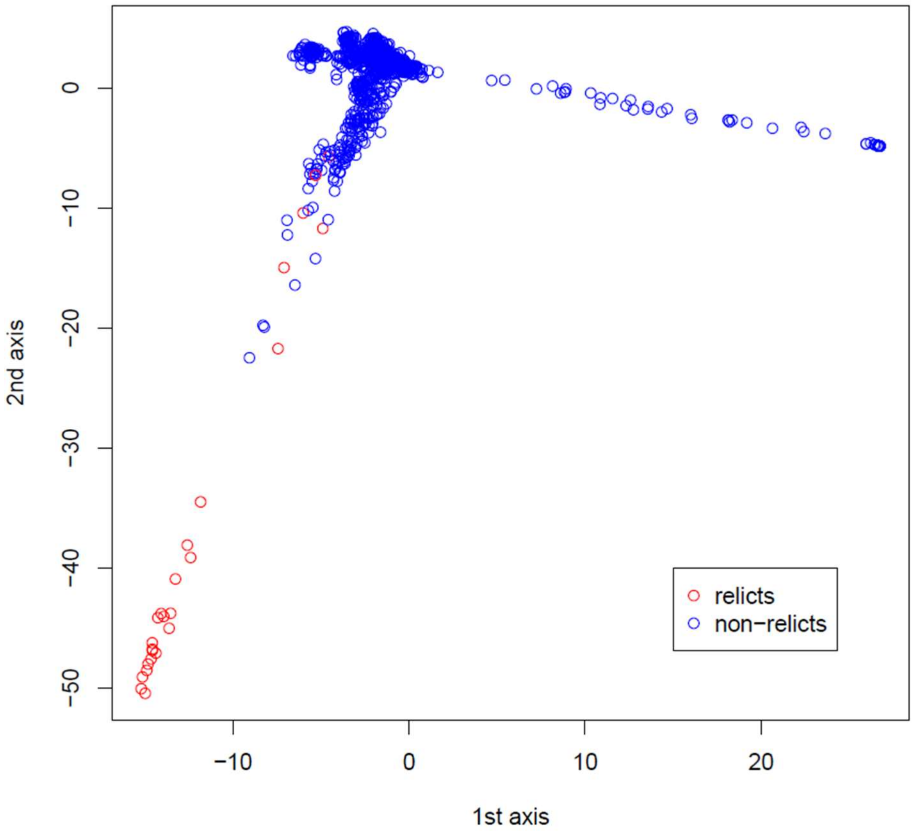

Figure S2 Multidimensional scaling of genotype data.

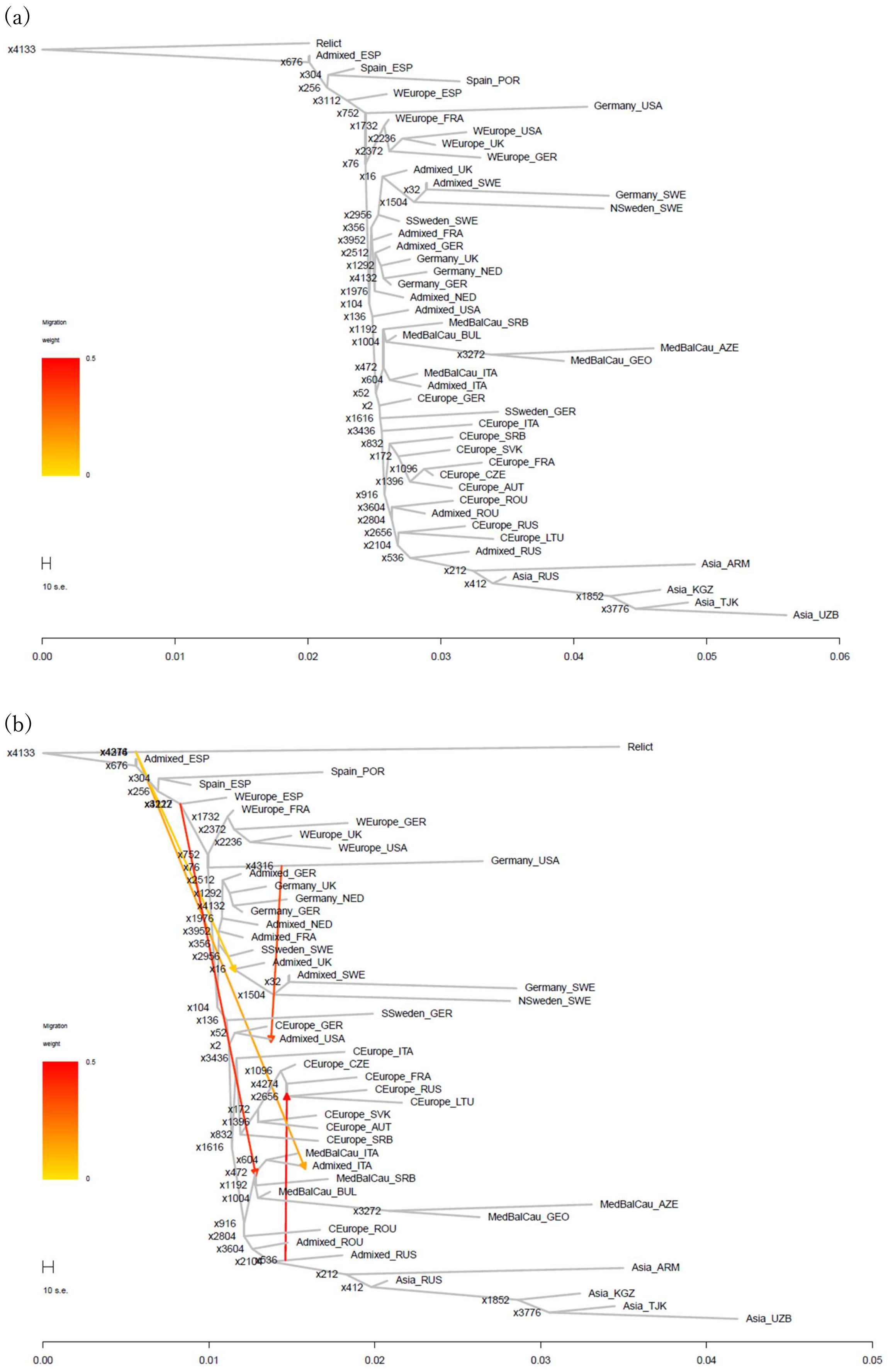
(a)
Relict
Admixed_ESP
Spain_ESP
Spain_POR
WEurope_ESP
Germany_USA
WEurope_FRA
WEurope_USA
WEurope_UK
WEurope_GER
Admixed_UK
Admixed_SWE
Germany_SWE
NSweden_SWE
SSweden_SWE
Admixed_FRA
Admixed_GER
Germany_UK
Germany_NED
Germany_GER
Admixed_NED
Admixed_USA
MedBalCau_SRB
MedBalCau_BUL
MedBalCau_AZE
MedBalCau_GEO
MedBalCau_ITA
Admixed_ITA
CEurope_GER
SSweden_GER
CEurope_ITA
CEurope_SRB
CEurope_SVK
CEurope_FRA
CEurope_CZE
CEurope_AUT
CEurope_ROU
Admixed_ROU
CEurope_RUS
CEurope_LTU
Admixed_RUS
Asia_ARM
Asia_RUS
Asia_KGZ
Asia_TJK
Asia_UZB
Migration weight
0.5
0
10 s.e.
0.00
0.01
0.02
0.03
0.04
0.05
0.06
(b)
Relict
Admixed_ESP
Spain_POR
Spain_ESP
WEurope_ESP
WEurope_FRA
WEurope_GER
WEurope_UK
WEurope_USA
Germany_USA
Admixed_GER
Germany_UK
Germany_NED
Germany_GER
Admixed_NED
Admixed_FRA
SSweden_SWE
Admixed_UK
Admixed_SWE
Germany_SWE
NSweden_SWE
SSweden_GER
CEurope_GER
Admixed_USA
CEurope_ITA
CEurope_CZE
CEurope_FRA
CEurope_RUS
CEurope_LTU
CEurope_SVK
CEurope_AUT
CEurope_SRB
MedBalCau_ITA
Admixed_ITA
MedBalCau_SRB
MedBalCau_BUL
MedBalCau_AZE
MedBalCau_GEO
CEurope_ROU
Admixed_ROU
Admixed_RUS
Asia_ARM
Asia_RUS
Asia_KGZ
Asia_TJK
Asia_UZB
Migration weight
0.5
0
10 s.e.
0.00
0.01
0.02
0.03
0.04
0.05

(c)

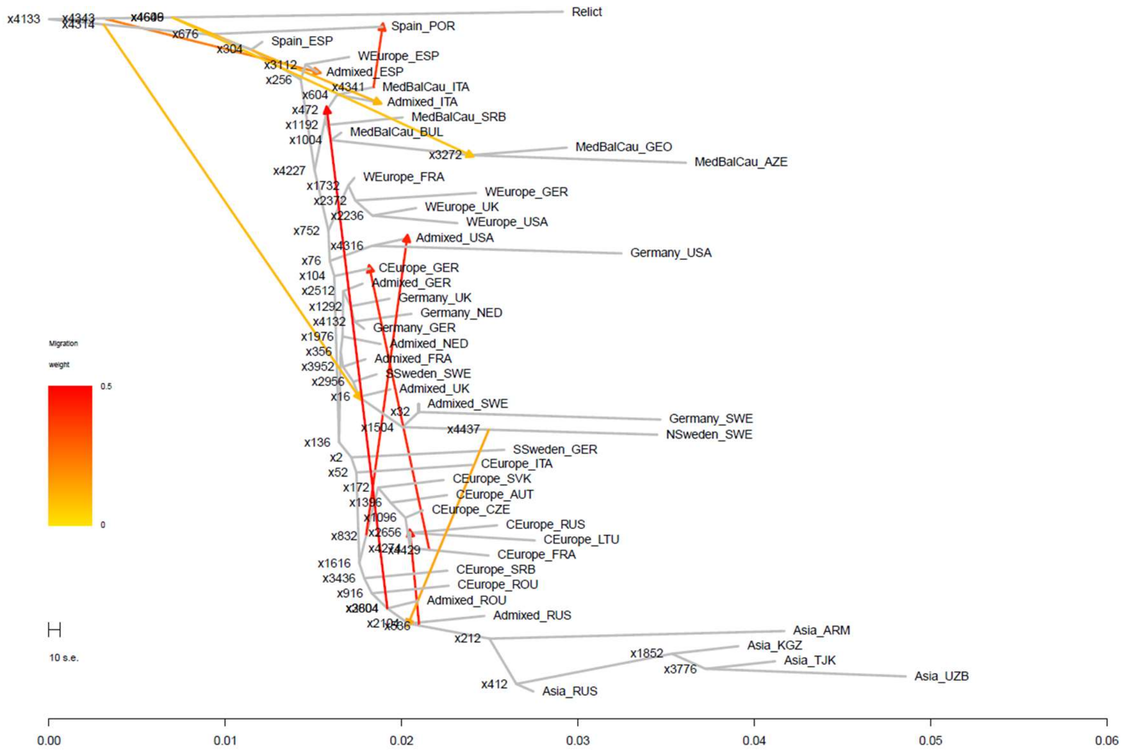

Figure S3 The admixture graph estimated by TreeMix. (a) no admixtures, (b) five admixtures, (c) ten admixtures. The terminal nodes are defined by the genetic clusters of ADMIXTURE (k=9, The 1001 Genomes Consortium 2016) subdivided by country (see Materials and Methods 2.2). The genetic clusters are labelled as Asia, Central Europe (CEurope), Germany, Italy-Balkan-Caucasus (ItaBalCau), Northern Sweden (NSweden), Southern Sweden (SSweden), Spain, and Western Europe (WEurope). The countries are labeled as Armenia (ARM), Austria (AUT), Azerbaijan (AZE), Bulgaria (BUL), Czech Republic (CZE), Spain (ESP), France (FRA), Georgia (GEO), Germany (GER), Italy (ITA), Kyrgyzstan (KGZ), Lithuania (LTU), Netherlands (NED), Portugal (POR), Romania (ROU), Russian Federation (RUS), Serbia (SRB), Switzerland (SUI), Slovakia (SVK), Sweden (SWE), Tajikistan (TJK), United Kingdom of Great Britain and Northern Ireland (UK), United States of America (USA), and Uzbekistan (UZB).

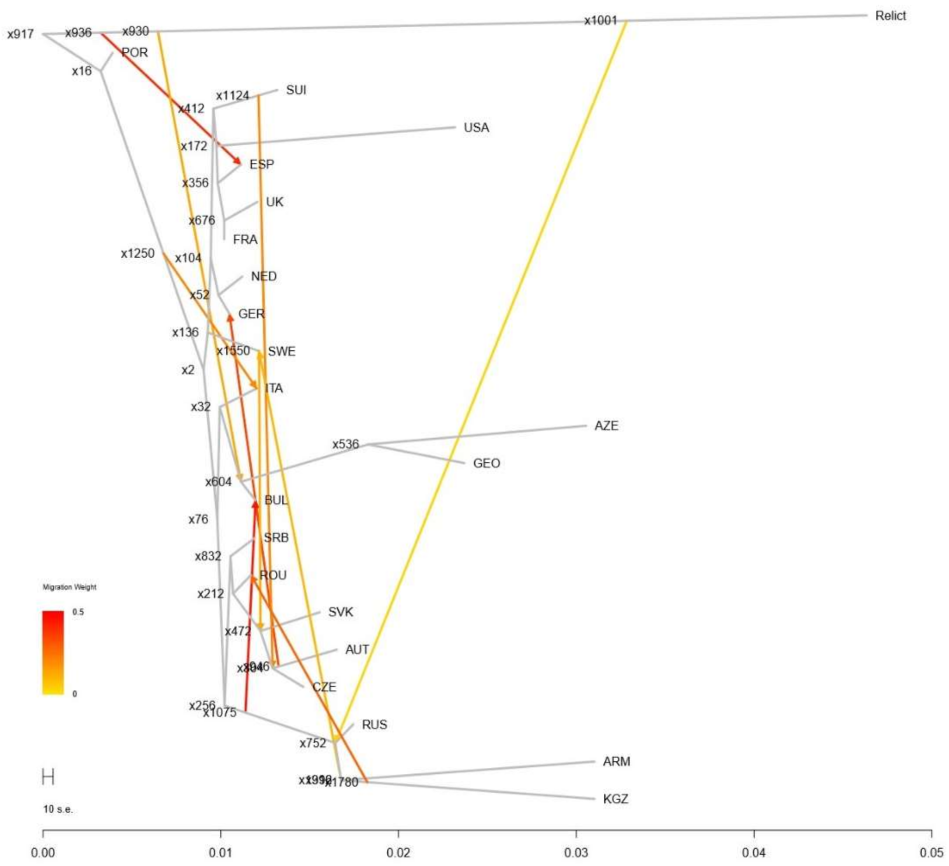

Figure S4 The admixture graph of the countries with ten admixtures. The countries are labeled as Armenia (ARM), Austria (AUT), Azerbaijan (AZE), Bulgaria (BUL), Czech Republic (CZE), Spain (ESP), France (FRA), Georgia (GEO), Germany (GER), Italy (ITA), Kyrgyzstan (KGZ), Lithuania (LTU), Netherlands (NED), Portugal (POR), Romania (ROU), Russian Federation (RUS), Serbia (SRB), Switzerland (SUI), Slovakia (SVK), Sweden (SWE), Tajikistan (TJK), United Kingdom of Great Britain and Northern Ireland (UK), United States of America (USA), and Uzbekistan (UZB).

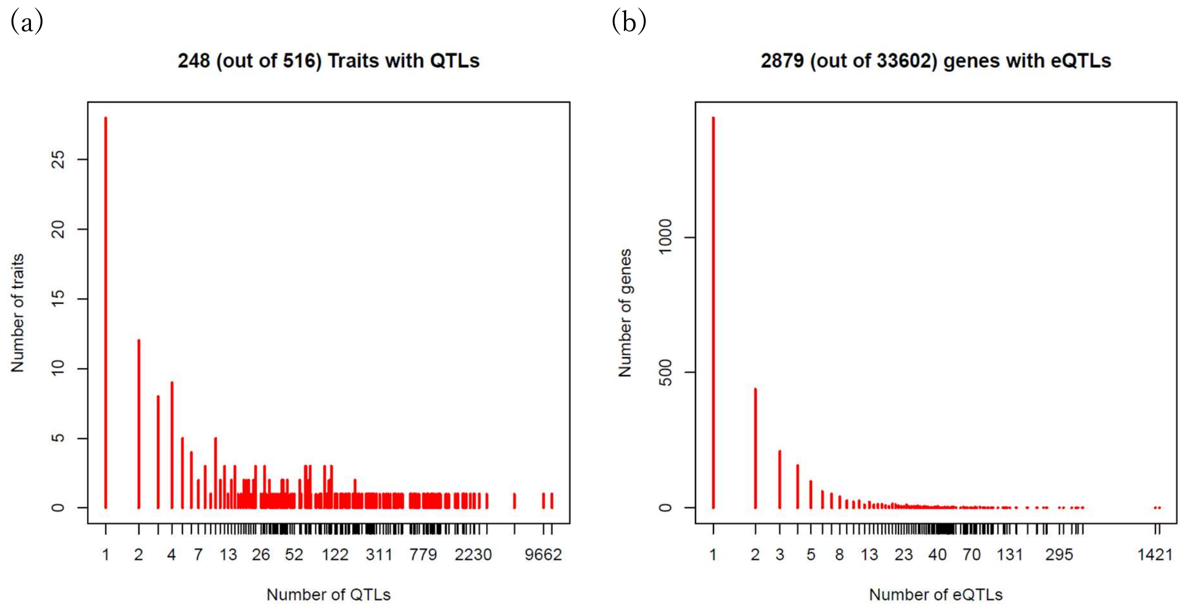

Figure S5 The distributions of numbers of QTLs and eQTLs. (a) The distribution of the number of QTLs among the traits with identified QTLs. Out of 516, 248 had identified QTLs with p-values less than 10^{-6}. (b) The distribution of the number of eQTLs among the gene expressions with identified eQTLs. Out of 33,602, 2,879 had identified eQTLs with p-values less than 10^{-6}. Note that the x-axis is log-scaled.

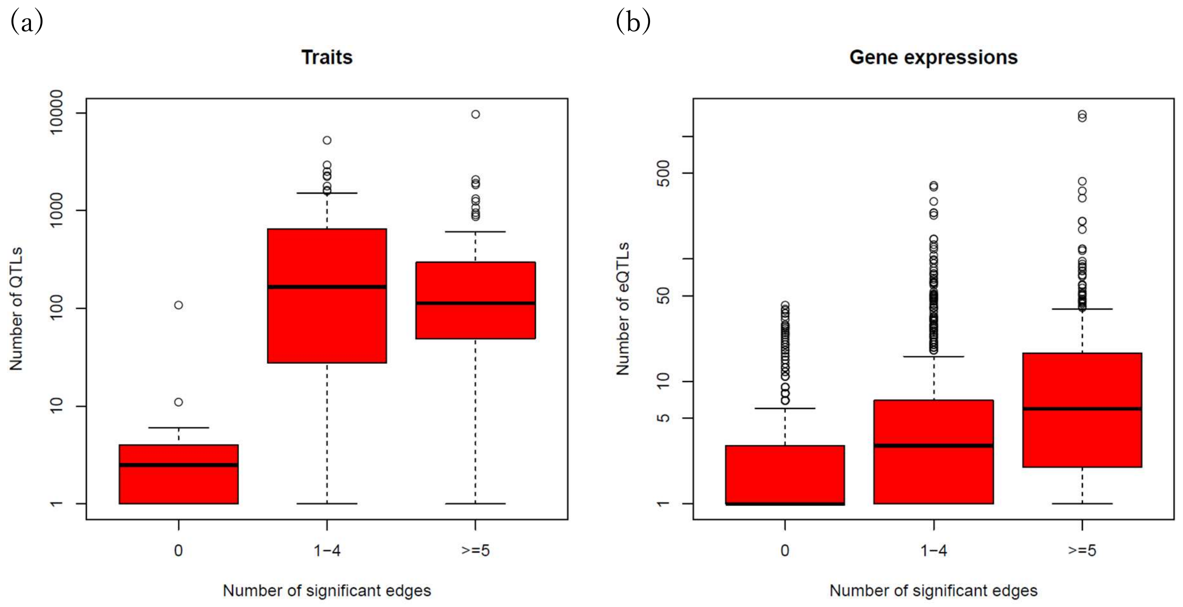

Figure S6 The traits and genes with many QTLs and eQTLs tend to have undergone multiple times p- and e-adaptations. The boxplots show the distributions of the numbers of QTLs of the traits (a) and of eQTLs of the gene expressions (b) for each category of numbers of edges with identified p- and e-adaptations. Note that the y-axis is log-scaled.

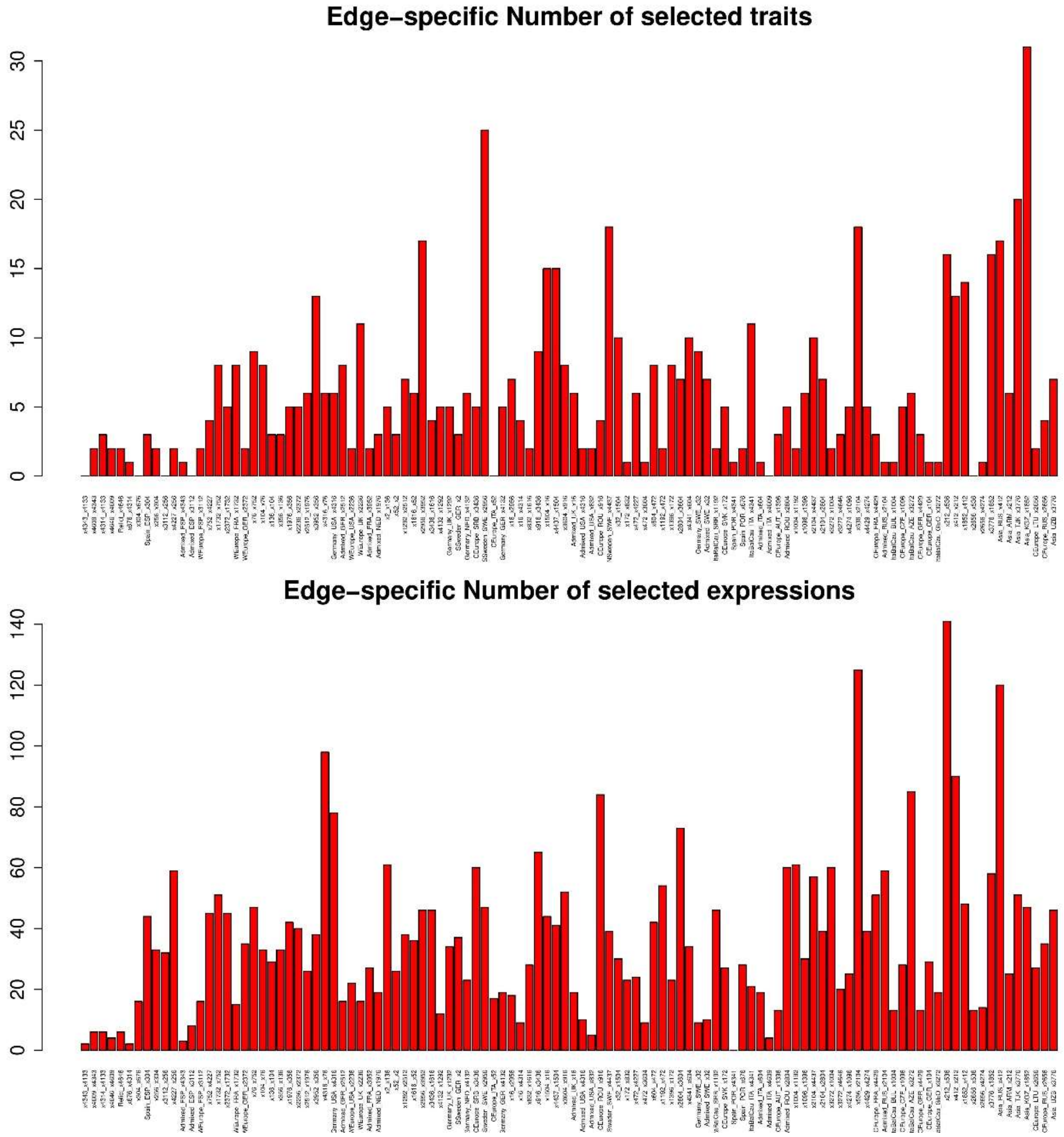

Figure S7 The numbers of p-adaptations and e-adaptations along the edges of the admixture graph. Bar plots representing the numbers of the adaptations are shown. The edges are labeled by the two nodes they connect (Figure S3). The exact numbers are shown in Table S3.

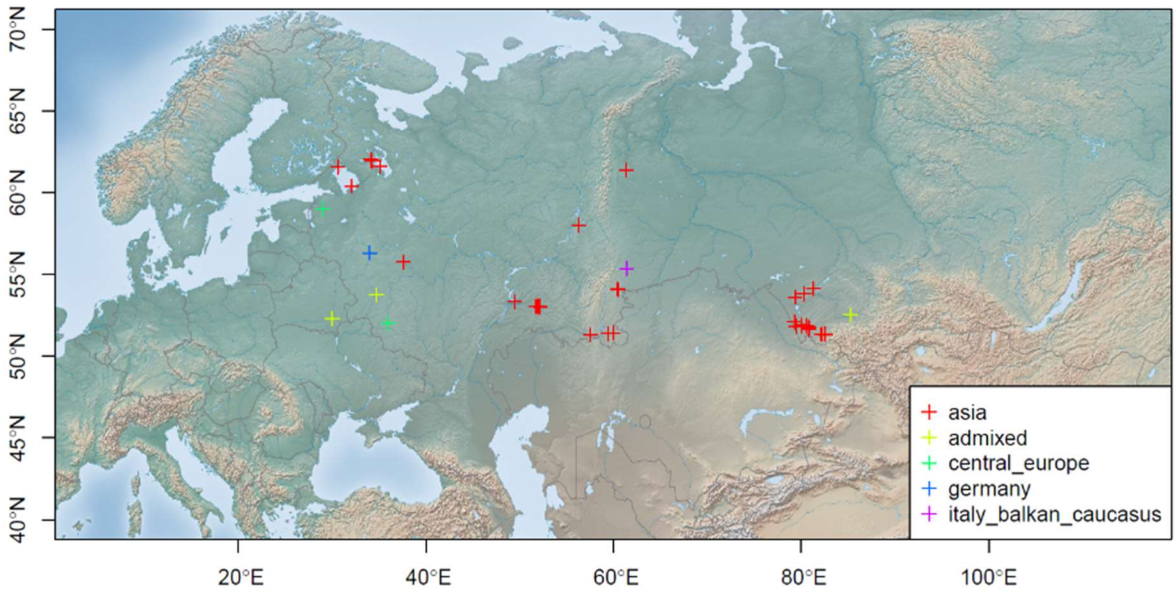

Figure S8 The assignment of the sample from Russia to the admixture groups.

(a)

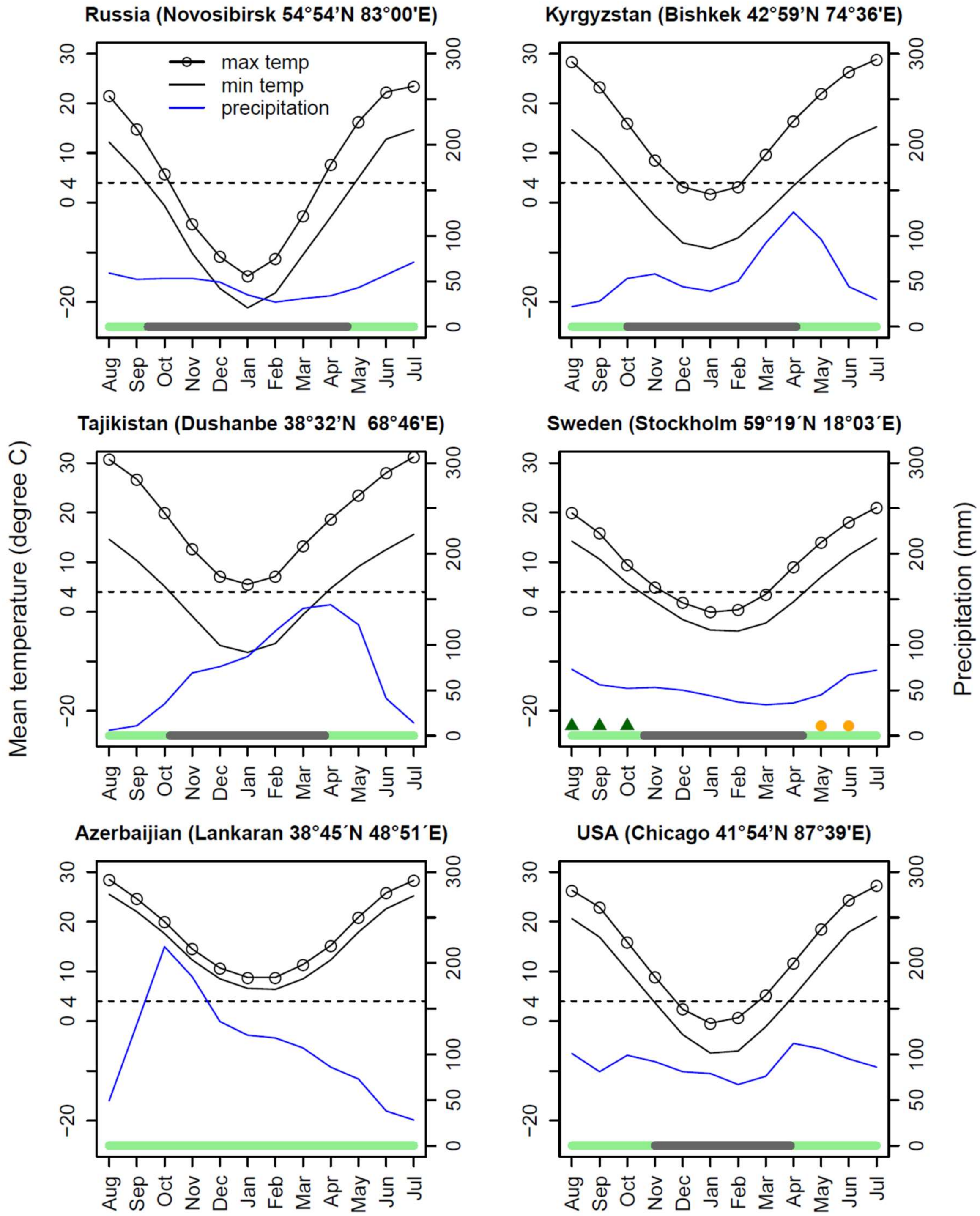
Russia (Novosibirsk 54°54'N 83°00'E)
Kyrgyzstan (Bishkek 42°59'N 74°36'E)
Tajikistan (Dushanbe 38°32'N 68°46'E)
Sweden (Stockholm 59°19´N 18°03´E)
Azerbaijian (Lankaran 38°45´N 48°51´E)
USA (Chicago 41°54'N 87°39'E)
max temp
min temp
precipitation
Mean temperature (degree C)
Precipitation (mm)
Aug
Sep
Oct
Nov
Dec
Jan
Feb
Mar
Apr
May
Jun
Jul

(b)

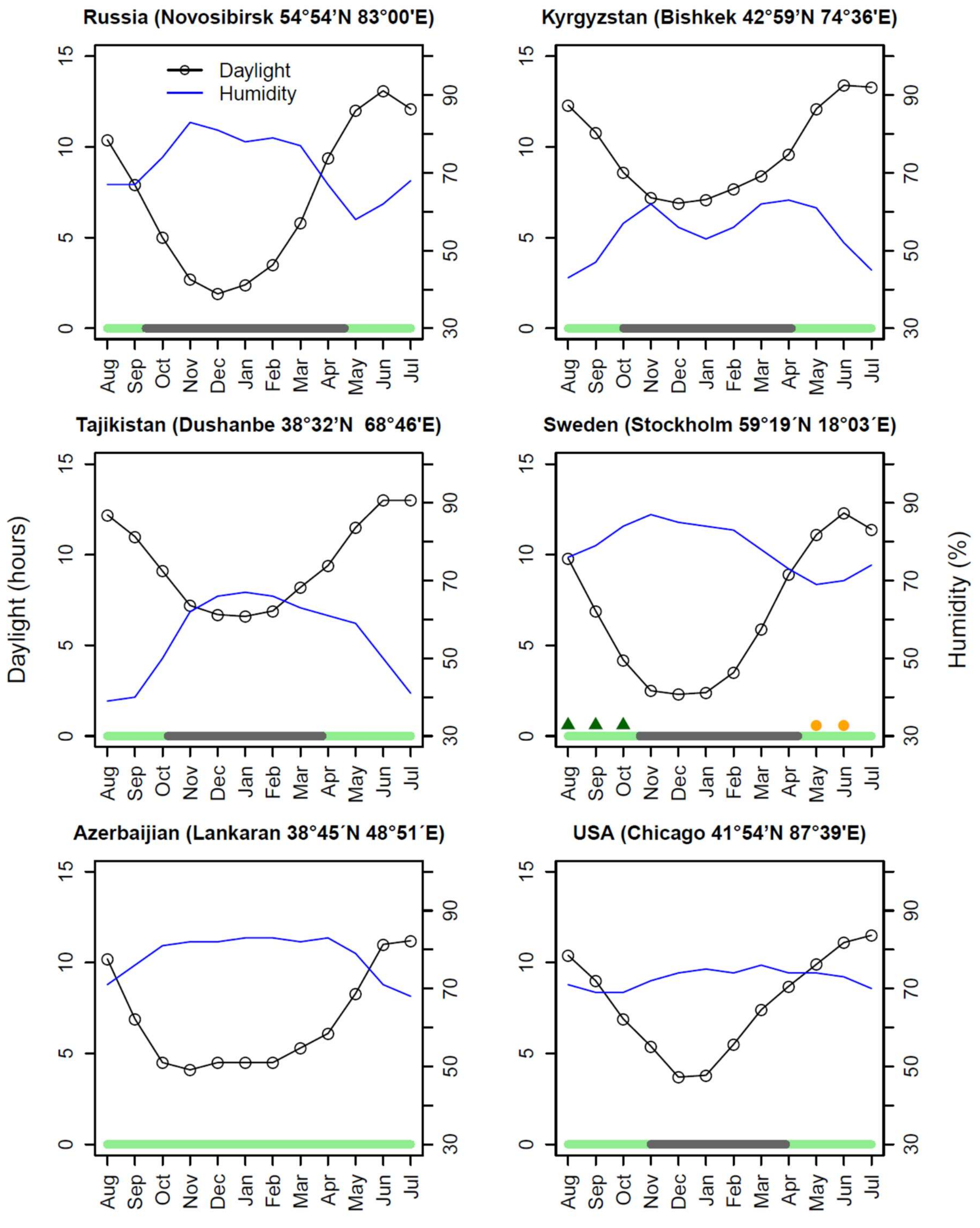

Figure S9 Climates of the cities representing the sampling locations from the four lineages with large scale adaptations. (a) Temperature and precipitation, (b) daylight and humidity. Climate Data. Accessed March 2023. Available: https://en.climate-data.org/. The dotted horizontal line shows the 4 ℃, the minimum monthly mean temperature required for growing season months (green bars on the bottom, Nordborg & Bergelson, 1999; Lasky et al., 2012). Green triangles and orange circles show seed germination and flowering times in Sweden (Ågren & Schemske, 2012).

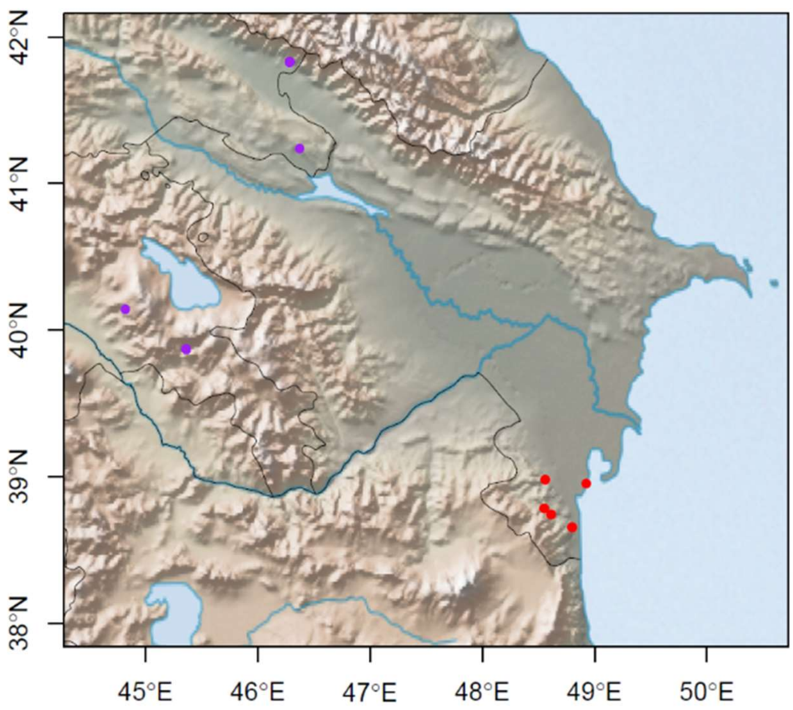

Figure S10 The sampling points in Azerbaijan. Points in red are in Azerbaijan, while points in purple are in Armenia and Georgia.

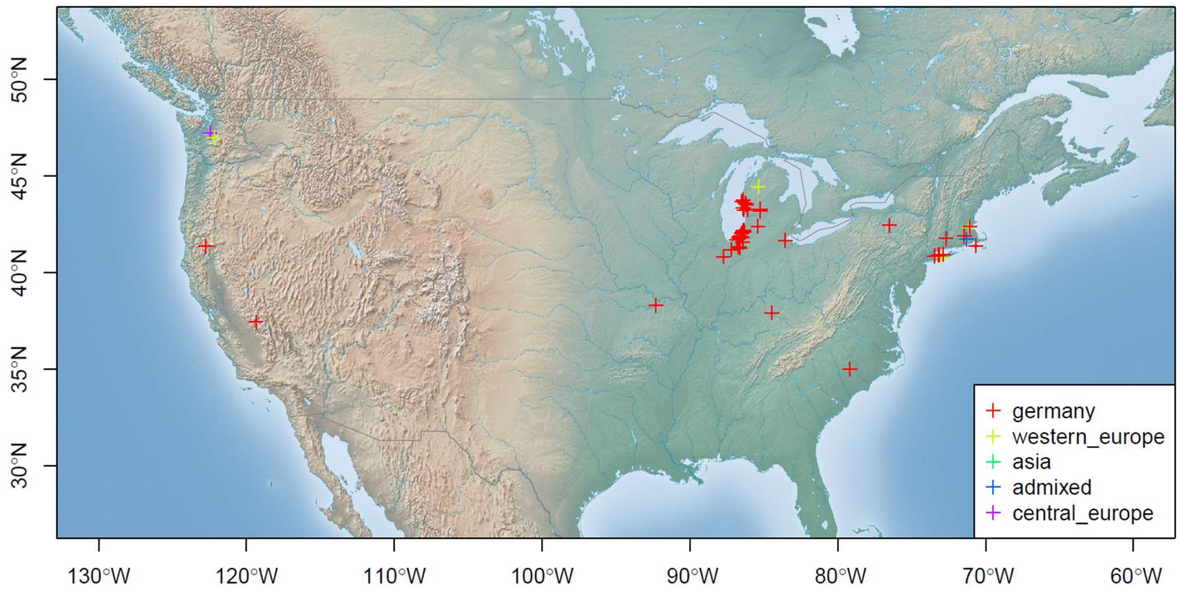

Figure S11 The assignment of the sample from the United States to the admixture groups.

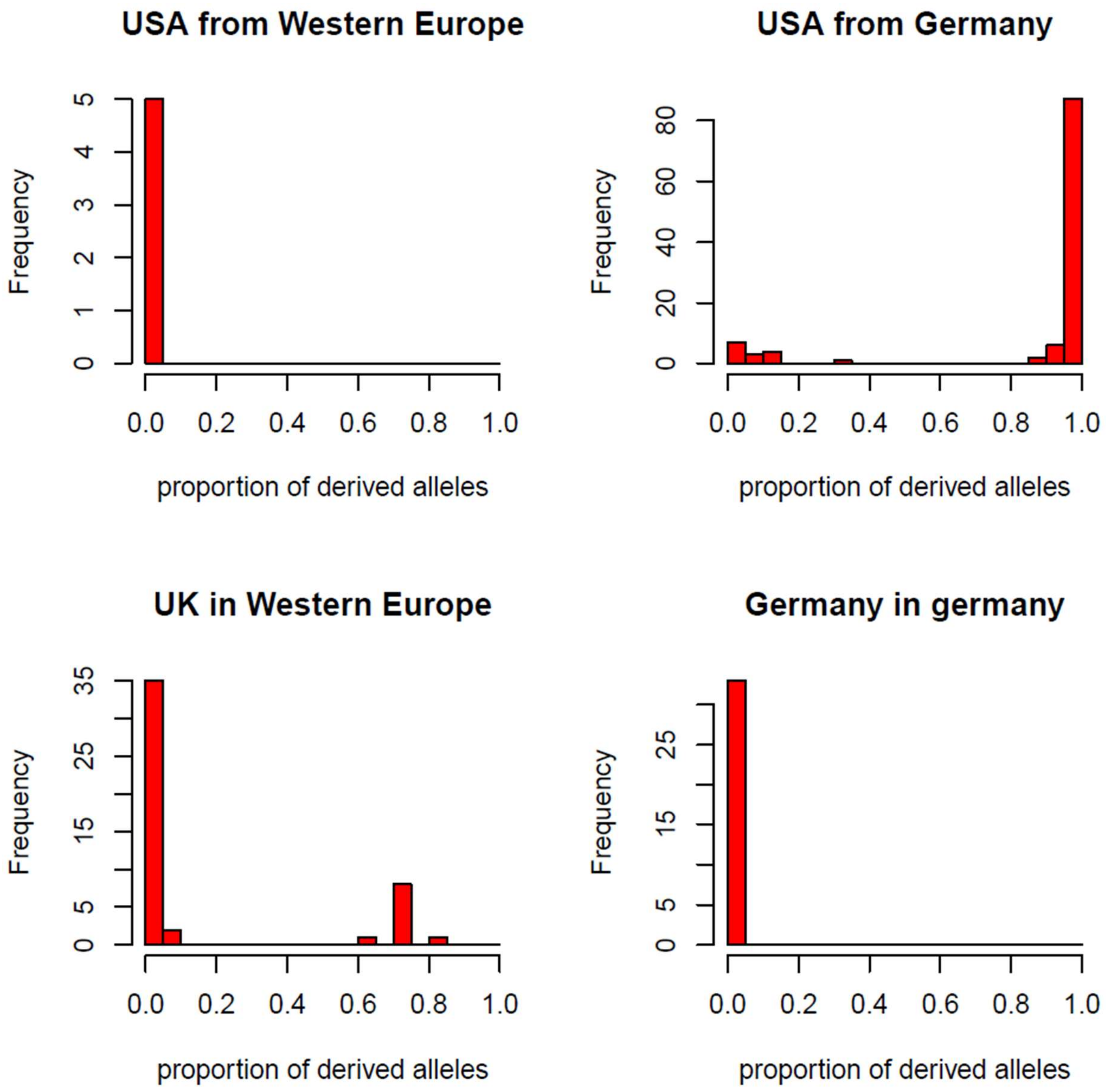

Figure S12 The proportions of the H-alleles at the photosynthesis-related eQTLs, whose allele frequencies changed significantly (FDR=0.05) along the lineage to the United States. The sample from the United States in the admixture groups of Western Europe and Germany, the sample from the United Kingdom of Great Britain in the admixture group of Western Europe, and the sample from Germany in the admixture groups of Germany are contrasted. The photosynthesis-related genes that were identified in the enrichment analysis of e-adaptations along the lineage to the United States and the eQTLs whose allele frequencies changed significantly (FDR=0.05) along this lineage were analyzed. Each eQTL has H-allele (higher expression) and L-allele (lower expression). For each individual, proportion of H-alleles were calculated. Here we called these alleles as derived alleles simply because they were minor alleles.

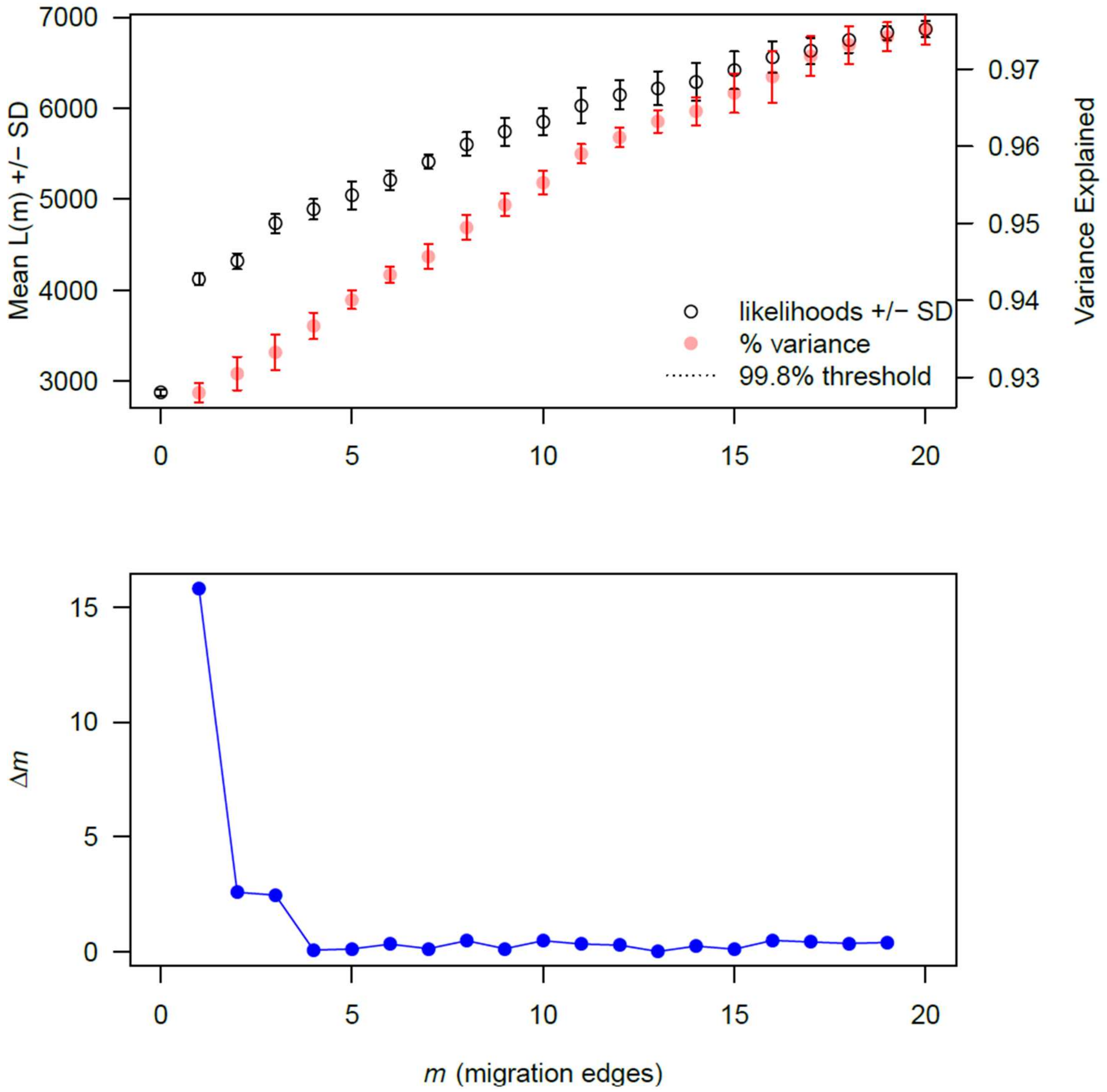

Figure S13 The output produced by OptM. (a) The mean and standard deviation (SD) across 10 iterations for the composite likelihood $L(m)$ (left axis, black circles) and proportion of variance explained (right axis, red "x"s). (b) The second-order rate of change ($\Delta(m)$) across values of m.

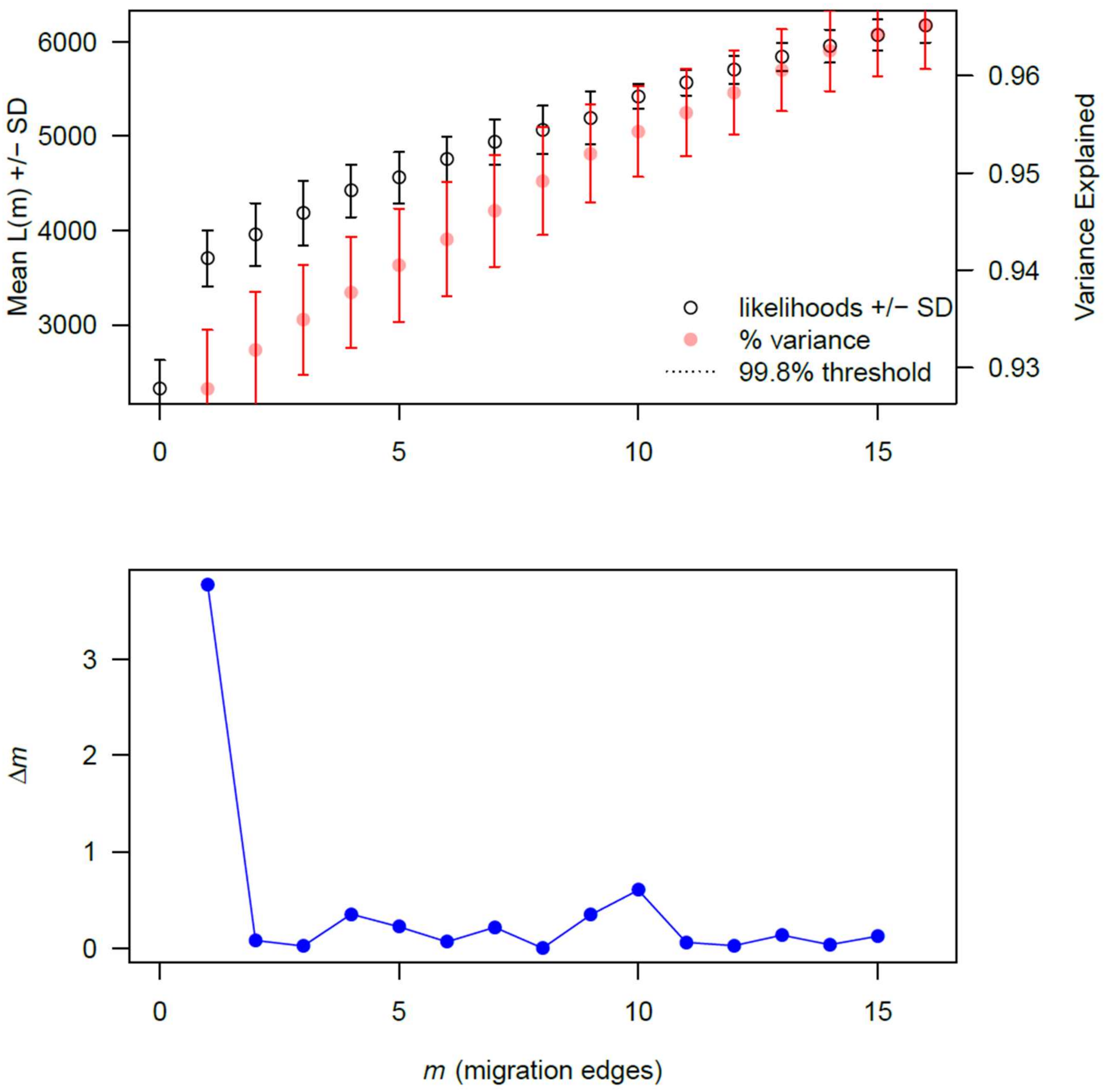

Figure S14 The output produced by OptM obtained by block resampling of genomic regions consisting of 300 SNPs. (a) The mean and standard deviation (SD) across 10 iterations for the composite likelihood $L(m)$ (left axis, black circles) and proportion of variance explained (right axis, red "x"s). (b) The second-order rate of change ($\Delta(m)$) across values of m.

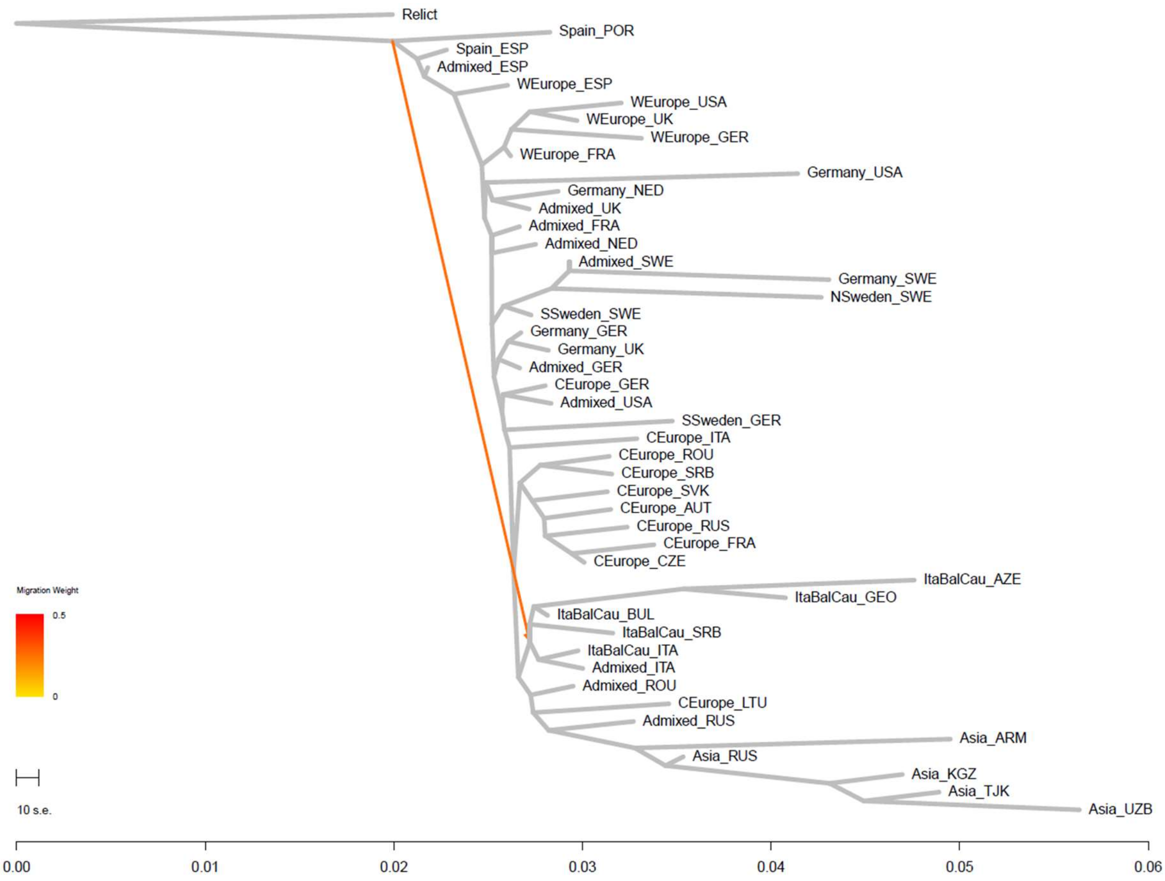

Figure S15 The admixture graph estimated by TreeMix, assuming the number of admixture edges, $m = 1$.

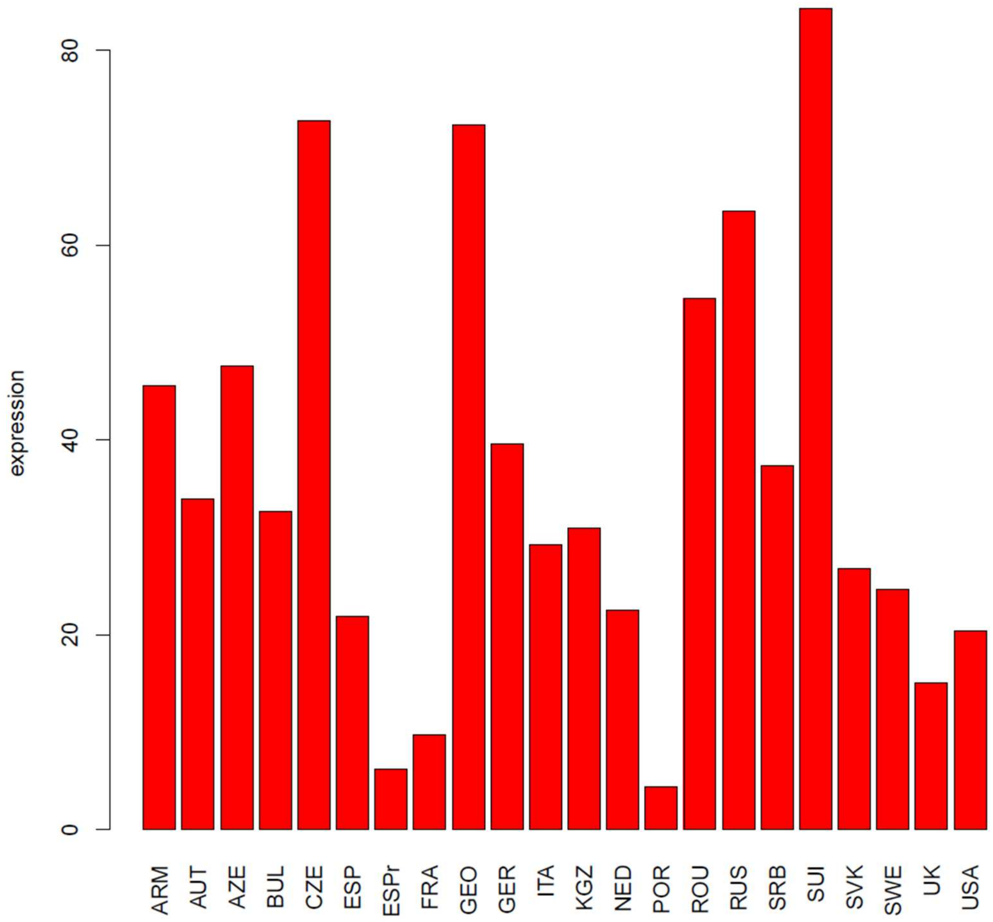

Figure S16 Variable mean expression levels of DOG1 among countries. The countries are labeled as Armenia (ARM), Austria (AUT), Azerbaijan (AZE), Bulgaria (BUL), Czech Republic (CZE), Spain (ESP), France (FRA), Georgia (GEO), Germany (GER), Italy (ITA), Kyrgyzstan (KGZ), Lithuania (LTU), Netherlands (NED), Portugal (POR), Romania (ROU), Russian Federation (RUS), Serbia (SRB), Switzerland (SUI), Slovakia (SVK), Sweden (SWE), Tajikistan (TJK), United Kingdom of Great Britain and Northern Ireland (UK), United States of America (USA), and Uzbekistan (UZB).

Table S1 Enrichment analysis of the genes with identified e-adaptations

category	term	p_value	fdr	description
Process	GO:000962	3.06E-13	1.44E-09	Response to abiotic stimulus
Process	GO:005089	3.01E-12	7.08E-09	Response to stimulus
Process	GO:000695	2.59E-10	4.06E-07	Response to stress
Process	GO:001003	6.80E-10	8.00E-07	Response to organic substance
Process	GO:004222	3.77E-09	3.55E-06	Response to chemical
Process	GO:190170	8.05E-09	6.31E-06	Response to oxygen-containing compound
Process	GO:000941	1.15E-07	7.73E-05	Response to light stimulus
Process	GO:000931	1.35E-07	7.92E-05	Response to radiation
Process	GO:001024	1.55E-07	8.11E-05	Response to organonitrogen compound
Process	GO:005171	2.41E-07	0.00011	Cellular response to stimulus
Process	GO:000166	3.35E-07	0.00012	Response to hypoxia
Process	GO:000695	3.02E-07	0.00012	Defense response
Process	GO:001020	3.24E-07	0.00012	Response to chitin
Process	GO:007088	2.70E-07	0.00012	Cellular response to chemical stimulus
Process	GO:007145	3.89E-07	0.00012	Cellular response to hypoxia
Process	GO:190169	2.90E-07	0.00012	Response to nitrogen compound
Process	GO:000237	1.02E-06	0.00023	Immune system process
Process	GO:000971	1.23E-06	0.00026	Response to endogenous stimulus
Process	GO:000972	1.42E-06	0.00029	Response to hormone
Process	GO:000981	2.59E-06	0.00051	Defense response, incompatible interaction
Process	GO:000940	6.29E-06	0.0012	Response to cold
Process	GO:000926	1.04E-05	0.0019	Response to temperature stimulus
Process	GO:004508	1.20E-05	0.0021	Innate immune response
Process	GO:000110	1.59E-05	0.0027	Response to acid chemical
Process	GO:000960	1.60E-05	0.0027	Response to biotic stimulus
Process	GO:005170	1.92E-05	0.0029	Response to other organism
Process	GO:000960	2.98E-05	0.0042	Response to external stimulus
Process	GO:000998	3.59E-05	0.005	Cellular process
Process	GO:003399	3.89E-05	0.0052	Response to lipid
Process	GO:004441	3.98E-05	0.0052	Interspecies interaction between organisms
Process	GO:003355	6.60E-05	0.0084	Cellular response to stress
Process	GO:000962	8.08E-05	0.01	Systemic acquired resistance

continued

Table S1 Enrichment analysis of the genes with identified e-adaptations (continued)

category	term	p_value	fdr	description
Process	GO:000941	9.39E-05	0.0113	Response to water
Process	GO:000941	0.00014	0.0159	Response to water deprivation
Process	GO:001022	0.00014	0.0159	Response to UV-B
Process	GO:009730	0.00014	0.0159	Response to alcohol
Process	GO:000973	0.00022	0.0238	Response to abscisic acid
Process	GO:000961	0.00024	0.0253	Response to bacterium
Process	GO:009854	0.00028	0.0297	Defense response to other organism
Process	GO:000268	0.00035	0.0353	Regulation of immune system process
Process	GO:005078	0.00035	0.0353	Regulation of biological process
Process	GO:000975	0.00038	0.0367	Hormone-mediated signaling pathway
Process	GO:006500	0.00038	0.0369	Biological regulation
Process	GO:000979	0.00047	0.0442	Post-embryonic development
Process	GO:001597	5.00E-04	0.0461	Photosynthesis
Process	GO:005079	5.00E-04	0.0461	Regulation of cellular process
Component	GO:000953	1.56E-06	0.00091	Photosystem II reaction center
Component	GO:011016	1.11E-06	0.00091	Cellular anatomical entity
Component	GO:000562	0.00013	0.0357	Intracellular
Component	GO:000952	0.00013	0.0357	Photosystem
Function	GO:004687	7.00E-06	0.0147	Metal ion binding

Table S2 Enrichment analysis of the genes with no identified e-adaptations

category	term	p_value	fdr	description
Process	GO:001003	4.57E-06	0.0159	Response to organic substance
Process	GO:005089	3.38E-06	0.0159	Response to stimulus
Process	GO:004222	1.28E-05	0.0201	Response to chemical
Function	GO:000382	4.98E-06	0.0105	Catalytic activity

Table S3 The numbers of p-adaptations and e-adaptations along each of the edges of the admixture graph. The edges are labeled by the two nodes they connect (Figure S3).

Edge	Number of p-adaptations	Number of e-adaptations	Edge	Number of p-adaptations	Number of e-adaptations	Edge	Number of p-adaptations	Number of e-adaptations
x4343_x4133	0	2	x1616_x52	6	36	Spain_POR_x676	2	28
x4609_x4343	2	6	x2956_x3952	17	46	ItaBalCau_ITA_x4341	11	21
x4314_x4133	3	6	x3436_x1616	4	46	Admixed_ITA_x604	1	19
x4646_x4609	2	4	x4132_x1292	5	12	Admixed_ITA_x4609	0	4
Relict_x4646	2	6	Germany_UK_x1292	5	34	CEurope_AUT_x1396	3	13
x676_x4314	1	2	SSweden_GER_x2	3	37	Admixed_ROU_x2804	5	60
x304_x676	0	16	Germany_NED_x4132	6	23	x1004_x1192	2	61
Spain_ESP_x304	3	44	CEurope_SRB_x3436	5	60	x1096_x1396	6	30
x256_x304	2	33	SSweden_SWE_x2956	25	47	x2104_x4437	10	57
x3112_x256	0	32	CEurope_ITA_x52	0	17	x2104_x2804	7	39
x4227_x256	2	59	Germany_GER_x4132	5	19	x3272_x1004	2	60
Admixed_ESP_x4343	1	3	x16_x2956	7	18	x3272_x4646	3	20
Admixed_ESP_x3112	0	8	x16_x4314	4	9	x4274_x1096	5	25
WEurope_ESP_x3112	2	16	x832_x1616	2	28	x536_x2104	18	125
x752_x4227	4	45	x916_x3436	9	65	x4429_x4274	5	39
x1732_x752	8	51	x1504_x16	15	44	CEurope_FRA_x4429	3	51
x2372_x1732	5	45	x4437_x1504	15	41	Admixed_RUS_x2104	1	59
WEurope_FRA_x1732	8	15	x3604_x916	8	52	ItaBalCau_BUL_x1004	1	13
WEurope_GER_x2372	2	35	Admixed_UK_x16	6	19	CEurope_CZE_x1096	5	28
x76_x752	9	47	Admixed_USA_x4316	2	10	ItaBalCau_AZE_x3272	6	85
x104_x76	8	33	Admixed_USA_x832	2	5	CEurope_GER_x4429	3	13
x136_x104	3	29	CEurope_ROU_x916	4	84	CEurope_GER_x104	1	29
x356_x136	3	33	NSweden_SWE_x4437	18	39	ItaBalCau_GEO_x3272	1	19
x1976_x356	5	42	x32_x1504	10	30	x212_x536	16	141
x2236_x2372	5	40	x172_x832	1	23	x412_x212	13	90
x2512_x1976	6	26	x472_x4227	6	24	x1852_x412	14	48
x3952_x356	13	38	x472_x3604	1	9	x2656_x536	0	13
x4316_x76	6	98	x604_x472	8	42	x2656_x4274	1	14
Germany_USA_x4316	6	78	x1192_x472	2	54	x3776_x1852	16	58
Admixed_GER_x2512	8	16	x1396_x172	8	23	Asia_RUS_x412	17	120
WEurope_USA_x2236	2	22	x2804_x3604	7	73	Asia_ARM_x212	6	25
WEurope_UK_x2236	11	16	x4341_x604	10	34	Asia_TJK_x3776	20	51
Admixed_FRA_x3952	2	27	Germany_SWE_x32	9	9	Asia_KGZ_x1852	31	47
Admixed_NED_x1976	3	19	Admixed_SWE_x32	7	10	CEurope_LTU_x2656	2	27
x2_x136	5	61	ItaBalCau_SRB_x1192	2	46	CEurope_RUS_x2656	4	35
x52_x2	3	26	CEurope_SVK_x172	5	27	Asia_UZB_x3776	7	46
x1292_x2512	7	38	Spain_POR_x4341	1	0	sum	650	3925

Table S4 p-adaptations and enrichment analysis of e-adaptations along the lineage to Central Asia and South Siberia, Russia

p-adaptations

id	trait	description
pid144	M216T665	metabolite content trait
pid145	M130T666	metabolite content trait
pid28	YEL	leaf chlorosis
pid360	Mean(LRR) C	root mass density
pid370	Mean(R) ABA	root branching
pid375	Mean(LRD R) ABA	root mass density
pid50	DSDS50	the days of seed dry storage required to reach 50% germination
pid549	clim-aet5	Actual evapotranspiration of May (mm)
pid550	clim-aet6	Actual evapotranspiration of June (mm)
pid587	clim-gs9	Growing season includes September (yes/no)
pid616	clim-tmax1	Maximum Temperature of January (_C)
pid618	clim-tmax11	Maximum Temperature of November (_C)
pid620	clim-tmax2	Maximum Temperature of Februrary (_C)
pid633	clim-tmin3	Minimum Temperature of March (_C)

enrichment analysis of e-adaptations

category	term	fdr	description
COMPARTMENTS	GOCC:0005576	0.0138	Extracellular region
Process	GO:0009627	0.0415	Systemic acquired resistance
Keyword	KW-0732	0.0089	Signal
NetworkNeighborAL	CL:21283	0.0032	Mixed, incl. systemic acquired resistance, and cellular response to salicylic acid stimulus
NetworkNeighborAL	CL:21392	0.0032	Mostly uncharacterized, incl. positive regulation of defense response to oomycetes, and proline-rich membrane anchor 1
NetworkNeighborAL	CL:21396	0.0093	Mixed, incl. proline-rich membrane anchor 1, and detection of molecule of fungal origin

Table S5 p-adaptations and enrichment analysis of e-adaptations along the lineage to Sweden

p-adaptations

id	trait	description
pid100	4W	days to flowering trait
pid102	FT22	days to flowering trait
pid104	SDV	days to flowering trait
pid11	2W	days to flowering trait
pid116	DTFplantingSummerLocSweden2009	days to flowering trait
pid117	DTFlocSweden2008	days to flowering trait
pid134	DTF sweden 2009 (1st experiment)	days to flowering trait
pid20	Storage 7 days	seed dormancy
pid261	FT10	days to flowering trait
pid326	GR63 cold	seed dormancy
pid49	FT GH	days to flowering trait
pid53	0W GH FT	days to flowering trait
pid671	Basal75	root morphology trait
pid87	LN16	leaf number
pid89	LD	days to flowering trait
pid99	8W GH FT	days to flowering trait

enrichment analysis of e-adaptations

category	term	fdr	description
COMPARTMENTS	GOCC:0031967	0.0138	Organelle envelope
COMPARTMENTS	GOCC:0005737	0.0443	Cytoplasm
COMPARTMENTS	GOCC:0009941	0.0443	Chloroplast envelope
Process	GO:0030029	0.0189	Actin filament-based process
Process	GO:0009644	0.025	Response to high light intensity
Process	GO:0030833	0.025	Regulation of actin filament polymerization
Process	GO:0051014	0.025	Actin filament severing
Process	GO:0007015	0.0266	Actin filament organization
Process	GO:0051693	0.0266	Actin filament capping
Process	GO:0010205	0.0281	Photoinhibition
Process	GO:0051017	0.0319	Actin filament bundle assembly
Process	GO:0010257	0.0338	NADH dehydrogenase complex assembly
Process	GO:0016043	0.0381	Cellular component organization
Process	GO:0009416	0.0422	Response to light stimulus
Process	GO:0055114	0.0429	Oxidation-reduction process
Process	GO:0008154	0.0459	Actin polymerization or depolymerization
Process	GO:0006996	0.0491	Organelle organization

continued

Table S5 p-adaptations and enrichment analysis of e-adaptations along the lineage to Sweden (continued)

enrichment analysis of e-adaptations

category	term	fdr	description
Process	GO:0022607	0.0491	Cellular component assembly
Component	GO:0015629	0.0326	Actin cytoskeleton
Component	GO:0031967	0.0326	Organelle envelope
Keyword	KW-0009	0.0168	Actin-binding
Keyword	KW-0117	0.0168	Actin capping
SMART	SM00153	0.0113	Villin headpiece domain
SMART	SM00262	0.0113	Gelsolin homology domain

Table S6 p-adaptations and enrichment analysis of e-adaptations along the lineage to Azerbaijan

p-adaptations

id	trait	description
pid592	clim-pet1	Potential evapotranspiration of January (mm)
pid595	clim-pet12	Potential evapotranspiration of December (mm)
pid605	clim-prec10	Precipitation of October (mm)

enrichment analysis of e-adaptations

category	term	fdr	description
Process	GO:0042221	5.38E-08	Response to chemical
Process	GO:0070887	5.38E-08	Cellular response to chemical stimulus
Process	GO:0071456	5.71E-08	Cellular response to hypoxia
Process	GO:0050896	1.17E-06	Response to stimulus
Process	GO:0010033	1.22E-06	Response to organic substance
Process	GO:0010200	2.29E-06	Response to chitin
Process	GO:0051716	2.40E-05	Cellular response to stimulus
Process	GO:0009628	7.67E-05	Response to abiotic stimulus
Process	GO:0010468	7.79E-05	Regulation of gene expression
Process	GO:0009755	0.00011	Hormone-mediated signaling pathway
Process	GO:1901700	0.00011	Response to oxygen-containing compound
Process	GO:2000112	0.00011	Regulation of cellular macromolecule biosynthetic process
Process	GO:0006950	0.00014	Response to stress
Process	GO:0009725	0.00021	Response to hormone
Process	GO:0051171	0.00046	Regulation of nitrogen compound metabolic process
Process	GO:0080090	0.00059	Regulation of primary metabolic process
Process	GO:0050794	0.0029	Regulation of cellular process
Process	GO:0009734	0.005	Auxin-activated signaling pathway
Process	GO:0009735	0.0051	Response to auxin
Process	GO:0009736	0.0064	Signal transduction
Process	GO:0009737	0.0071	Defense response
Process	GO:0009738	0.0119	Defense response to virus
Process	GO:0009739	0.0121	Response to other organism
Process	GO:0009740	0.0121	Defense response to other organism
Process	GO:0009741	0.0156	Response to ethylene
Process	GO:0009742	0.016	Biological regulation
Process	GO:0009743	0.0169	Olefinic compound metabolic process
Process	GO:0009744	0.0198	Fruit ripening
Process	GO:0009745	0.0206	Response to external stimulus
Process	GO:0009746	0.0206	Response to bacterium
Process	GO:0009747	0.0221	1-aminocyclopropane-1-carboxylate biosynthetic process
Process	GO:0009748	0.0238	Response to uv-b
Process	GO:0009749	0.0294	Response to water deprivation
Process	GO:0009750	0.0342	Response to wounding
Process	GO:0009751	0.0396	Ethylene-activated signaling pathway
Component	GO:0009752	0.0282	Nucleus

(continued)

Table S6 p-adaptations and enrichment analysis of e-adaptations along the lineage to Azerbaijan (continued)

enrichment analysis of e-adaptations

category	term	fdr	description
Function	GO:0009753	6.68E-06	DNA-binding transcription factor activity
Keyword	GO:0009754	9.36E-05	Transcription regulation
Keyword	GO:0009755	0.0092	Activator
Keyword	GO:0009756	0.0241	Fruit ripening
Keyword	GO:0009757	0.0252	DNA-binding
Keyword	GO:0009758	0.0319	Ethylene biosynthesis
Keyword	GO:0009759	0.0319	Nucleus
Keyword	GO:0009760	0.0319	Plant defense
Keyword	GO:0009761	0.0319	Auxin signaling pathway
Keyword	GO:0009762	0.0319	Ethylene signaling pathway
KEGG	GO:0009763	0.036	Plant hormone signal transduction
SMART	GO:0009764	0.00027	DNA-binding domain in plant proteins such as APETALA2 and EREBPs
InterPro	GO:0009765	0.003	AP2/ERF domain
InterPro	GO:0009766	0.003	DNA-binding domain superfamily
InterPro	GO:0009767	0.003	AP2/ERF domain superfamily
InterPro	GO:0009768	0.048	AUX/IAA protein
Pfam	GO:0009769	0.0014	AP2 domain
NetworkNeighborAL	GO:0009770	1.75E-19	Response to chitin, and cellular response to hypoxia
NetworkNeighborAL	GO:0009771	5.74E-18	Response to chitin, and cellular response to hypoxia
NetworkNeighborAL	GO:0009772	1.29E-15	Response to chitin, and cellular response to hypoxia
NetworkNeighborAL	GO:0009773	1.73E-11	Response to chitin, and cysteine-rich transmembrane cystm domain
NetworkNeighborAL	GO:0009774	5.97E-10	Response to chitin, and cellular response to hypoxia
NetworkNeighborAL	GO:0009775	4.78E-09	Response to chitin, and mac/perforin domain
NetworkNeighborAL	GO:0009776	5.81E-09	Domain of unknown function duf1117, and response to chitin
NetworkNeighborAL	GO:0009777	1.07E-05	Calmodulin binding protein-like, and caveola
NetworkNeighborAL	GO:0009778	0.00015	Regulation of tryptophan metabolic process, and response to ozone
NetworkNeighborAL	GO:0009779	0.0012	Domain of unknown function duf1117, and multicellular organism growth
NetworkNeighborAL	GO:0009780	0.0016	AUX/IAA family, and auxin binding
NetworkNeighborAL	GO:0009781	0.0228	Calmodulin binding protein-like, and negative regulation of defense response to bacterium
NetworkNeighborAL	GO:0009782	0.0228	Domain of unknown function duf1117, and multicellular organism growth
NetworkNeighborAL	GO:0009783	0.0332	AUX/IAA family
NetworkNeighborAL	GO:0009784	0.0477	Regulation of timing of plant organ formation, and floral organ senescence

Table S7 p-adaptations and enrichment analysis of e-adaptations along the lineage to the United States

p-adaptations

id	trait	description
pid17	avrRpm1	bacterial disease resistance
pid287	Cd111	cadmium concentration
pid37	0W	days to flowering trait
pid48	8W	days to flowering trait
pid58	LFS GH	reproductive growth time
pid8	Leaf roll 16	rolled leaf
pid88	avrB	bacterial disease resistance

enrichment analysis of e-adaptations

category	term	fdr	description
COMPARTMENTS	GOCC:0009579	8.07E-06	Thylakoid
COMPARTMENTS	GOCC:0034357	8.45E-06	Photosynthetic membrane
COMPARTMENTS	GOCC:0009535	2.61E-05	Chloroplast thylakoid membrane
COMPARTMENTS	GOCC:0009536	0.00029	Plastid
COMPARTMENTS	GOCC:0009507	0.00045	Chloroplast
COMPARTMENTS	GOCC:0009521	0.008	Photosystem
COMPARTMENTS	GOCC:0010287	0.0121	Plastoglobule
COMPARTMENTS	GOCC:0009512	0.0255	Cytochrome b6f complex
Process	GO:0015979	1.02E-09	Photosynthesis
Component	GO:0009539	1.05E-10	Photosystem ii reaction center
Component	GO:0009535	1.85E-10	Chloroplast thylakoid membrane
Component	GO:0009521	8.40E-09	Photosystem
Component	GO:0009507	1.54E-08	Chloroplast
Component	GO:0009536	2.67E-08	Plastid
Component	GO:0098796	1.91E-05	Membrane protein complex
Component	GO:0045259	0.0078	Proton-transporting atp synthase complex
Component	GO:0010287	0.0098	Plastoglobule
Component	GO:0009512	0.0131	Cytochrome b6f complex
Component	GO:0045261	0.0499	Proton-transporting atp synthase complex, catal
Function	GO:0009055	0.0081	Electron transfer activity
Function	GO:0045158	0.0081	Electron transporter, transferring electrons wit
Keyword	KW-0602	5.45E-13	Photosynthesis
Keyword	KW-0793	9.28E-11	Thylakoid
Keyword	KW-0150	7.41E-10	Chloroplast
Keyword	KW-0604	1.85E-07	Photosystem II
Keyword	KW-0674	2.64E-06	Reaction center
Keyword	KW-0691	3.71E-05	RNA editing
Keyword	KW-0375	0.00034	Hydrogen ion transport
Keyword	KW-0249	7.00E-04	Electron transport

(continued)

Table S7 p-adaptations and enrichment analysis of e-adaptations along the lineage to the United States (continued)

enrichment analysis of e-adaptations

category	term	fdr	description
Keyword	KW-0066	0.0037	ATP synthesis
Keyword	KW-0139	0.0491	CF(1)
KEGG	ath00195	1.90E-17	Photosynthesis
KEGG	ath00190	0.0171	Oxidative phosphorylation
InterPro	IPR036121	0.0308	ATPase, F1/V1/A1 complex, alpha/beta subunit, N-terminal domain superfamily
InterPro	IPR000194	0.0364	ATPase, F1/V1/A1 complex, alpha/beta subunit, nucleotide-binding domain
InterPro	IPR004100	0.0364	ATPase, F1/V1/A1 complex, alpha/beta subunit, N-terminal domain
InterPro	IPR020003	0.0364	ATPase, alpha/beta subunit, nucleotide-binding domain, active site
Pfam	PF00006	0.0379	ATP synthase alpha/beta family, nucleotide-binding domain
Pfam	PF00306	0.0379	ATP synthase alpha/beta chain, C terminal domain
Pfam	PF02874	0.0379	ATP synthase alpha/beta family, beta-barrel domain
NetworkNeighborAL	CL:12286	7.04E-24	Photosynthesis, and Plant protein of unknown function (DUF825)
NetworkNeighborAL	CL:12289	5.02E-23	Photosynthesis, and Plant protein of unknown function (DUF825)
NetworkNeighborAL	CL:12290	4.12E-20	Photosystem ii reaction center, and photosynthesis
NetworkNeighborAL	CL:12328	3.07E-13	Photosystem ii reaction center, and photosynthetic reaction centre, l/m
NetworkNeighborAL	CL:12331	3.83E-08	Photosystem ii reaction center, and photosynthetic reaction centre, l/m
NetworkNeighborAL	CL:12291	2.08E-05	Mixed, incl. atp synthesis, and plant protein of unknown function (duf825)
NetworkNeighborAL	CL:12312	0.002	ATP synthesis

Table S8 Enrichment analysis of the QTL-coding genes of the cadmium concentrations in leaves (Cd111)

category	term	inputGenes	preferred Names	fdr	description
Function	GO:0015662	AT4G30110, AT4G30120, AT4G30190	HMA2, HMA3, HA2	0.0031	Ion transmembrane transporter activity, phosphorylative mechanism
Function	GO:0016463	AT4G30110, AT4G30120	HMA2, HMA3	0.0034	Zinc transmembrane transporter activity, phosphorylative mechanism
Function	GO:0019829	AT4G30110, AT4G30120, AT4G30190	HMA2, HMA3, HA2	0.0034	ATPase-coupled cation transmembrane transporter activity
Function	GO:0015086	AT4G30110, AT4G30120	HMA2, HMA3	0.0129	Cadmium ion transmembrane transporter activity
Function	GO:0005385	AT4G30110, AT4G30120	HMA2, HMA3	0.0293	Zinc ion transmembrane transporter activity

Reference for Supplementary information

Ågren, J., & Schemske, D. W. (2012). Reciprocal transplants demonstrate strong adaptive differentiation of the model organism *Arabidopsis thaliana* in its native range. New Phytologist, 194, 1112–1122. https://doi.org/10.1111/j.1469-8137.2012.04112.x

Lasky, J. R., Des Marais, D. L., Mckay, J. K., Richards, J. H., Juenger, T. E., & Keitt, T. H. (2012). Characterizing genomic variation of *Arabidopsis thaliana*: the roles of geography and climate. Molecular Ecology. 21, 5512-5529. https://doi.org/10.1111/j.1365-294X.2012.05709.x

Nordborg, M. & Bergelson, J. (1999). The effect of seed and rosette cold treatment on germination and flowering time in some *Arabidopsis thaliana* (Brassicaceae) ecotypes. American Journal of Botany 86, 470–475. https://doi.org/10.2307/2656807
